# RestraintMaker: a graph-based approach to select distance restraints in free-energy calculations with dual topology

**DOI:** 10.1007/s10822-022-00445-6

**Published:** 2022-03-22

**Authors:** Benjamin Ries, Salomé Rieder, Clemens Rhiner, Philippe H. Hünenberger, Sereina Riniker

**Affiliations:** Laboratory of Physical Chemistry, ETH Zürich, Vladimir-Prelog-Weg 2, Zürich, 8093 Switzerland

**Keywords:** Molecular dynamics, Free energy calculation, Protein-ligand binding, Topology

## Abstract

The calculation of relative binding free energies (RBFE) involves the choice of the end-state/system representation, of a sampling approach, and of a free-energy estimator. System representations are usually termed “single topology” or “dual topology”. As the terminology is often used ambiguously in the literature, a systematic categorization of the system representations is proposed here. In the dual-topology approach, the molecules are simulated as separate molecules. Such an approach is relatively easy to automate for high-throughput RBFE calculations compared to the single-topology approach. Distance restraints are commonly applied to prevent the molecules from drifting apart, thereby improving the sampling efficiency. In this study, we introduce the program RestraintMaker, which relies on a greedy algorithm to find (locally) optimal distance restraints between pairs of atoms based on geometric measures. The algorithm is further extended for multi-state methods such as enveloping distribution sampling (EDS) or multi-site $\lambda$-dynamics. The performance of RestraintMaker is demonstrated for toy models and for the calculation of relative hydration free energies. The Python program can be used in script form or through an interactive GUI within PyMol. The selected distance restraints can be written out in GROMOS or GROMACS file formats. Additionally, the program provides a human-readable JSON format that can easily be parsed and processed further. The code of RestraintMaker is freely available on GitHub *https://github.com/rinikerlab/restraintmaker*.

**Supplementary Information:**

The online version contains supplementary material available at 10.1007/s10822-022-00445-6.

## Introduction

Recent methodological developments have improved the statistical robustness and the degree of automation of relative binding free energy (RBFE) calculations, which are now routinely applied in drug discovery projects in industry [1–13].

A free-energy calculation provides information about the relative populations of multiple end-states in equilibrium. Examples are found in drug design, where the end-states represent the different ligands that bind to a protein [8, 14–22], or in protein engineering, where the end-states correspond to the different amino acids considered for one position in the protein [23–25]. Each free-energy calculation involves the choice of a sampling approach, a free-energy estimator (e.g. thermodynamic integration (TI) [26], the Zwanzig equation [27], or Bennett’s acceptance ratio (BAR) [28]), and a representation of the end-states (i.e., molecules or substructures of molecules) during the simulation.

**Fig. 1:**
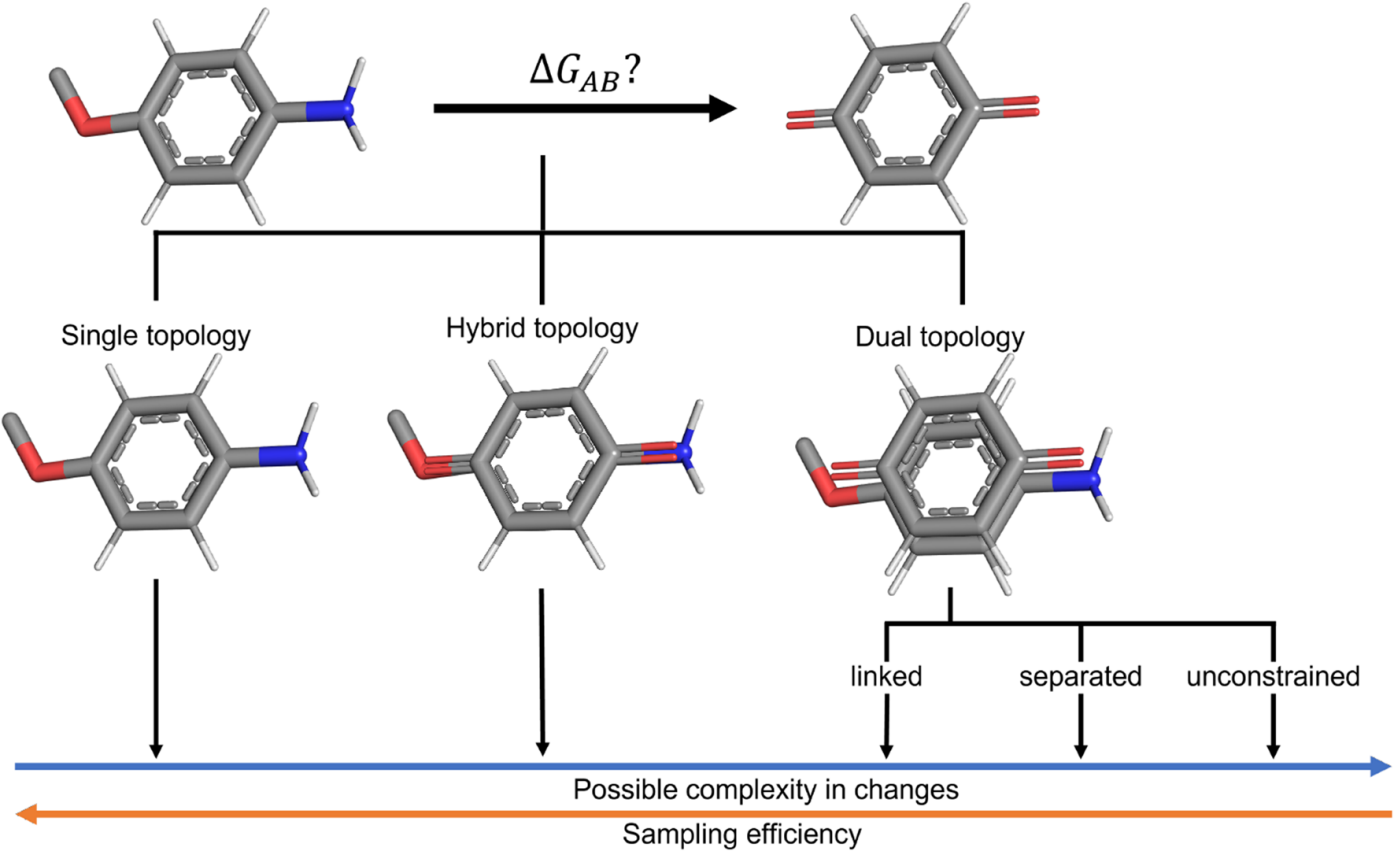
Three end-state representations can be distinguished based on the coordinate space. The “single-topology” approach (left) contains a single set of coordinates for all end-states. The “dual-topology” approach contains separate sets of coordinates for each end-state (right). The “hybrid-topology” approach (middle) combines atoms of common substructures into one coordinate set, while atoms that differ between the end-states are represented separately. It is therefore an intermediate between the two other representations. The dual topology approach can be further subdivided into three sub-variants: linked, separated, and unconstrained. The linked dual-topology approach is closest to the single topology approach, as the coordinate overlap between the end-states is enforced using direct spatial restraints (e.g. distance restraints). The separated variant is connecting the molecules indirectly by restraining them spatially to the same area, whereas the unconstrained variant does not restrain the molecules at all and is therefore also the most difficult to sample.

Several possible representations have been proposed in the past to build a coordinate and topology space of the end-states. Historically, two approaches emerged, which were termed “single topology” [29, 30] and “dual topology” [29, 31]. Unfortunately, the terminology is not always clear in the literature and these terms are used ambiguously [32–34]. To distinguish the different representation options, we propose here a terminology based on the difference in the respective coordinate space (Fig. 1). These definitions may differ from the historical ones. The single-topology approach contains a single set of coordinates for both end-states. In contrast, the dual-topology approach involves a separate set of coordinates for each end-state. The two approaches can be seen as opposite extremes. Three sub-variants of the dual-topology approach can be found in the literature: linked, separated and unconstrained. In addition, a “hybrid-topology” approach was recently described [21], which presents an intermediate between the single and dual approaches (Fig. 1). This scheme has been used in many studies for binding free energy calculations previously but not called hybrid topology. In protein engineering, Shobana et al. [23] called a similar approach hybrid topology. The different representations vary with respect to sampling efficiency and the capability of handling complex transformations.

With the high-throughput application of RBFE calculations comes the need for automation [10]. While there exist tools such as FESetup [35], ProtoCaller [36], SMArt [37], or LOMAP [38] to automatically set up single-topology RBFE calculations, the dual-topology approaches are in principle the easiest to automate as any alchemical molecule transformation can be realized without requiring atom mapping [33]. For the unconstrained dual-topology variant, an automatic set-up procedure is available in the packages pyAutoFEP [39] and FEW [40]. When representing the end-states with a linked dual-topology approach, the set-up is more difficult than in the unconstrained case as the distance restraints between the molecules need to be chosen. For example, the QligFEP pipeline [8] provides an automatic system generation for the linked dual-topology approach, where the distance restraints are placed in the perturbed common substructure of the end-states. These distance restraints only become active if the distance between the restrained atoms exceeds 0.02  nm. However, for complex transformations (e.g. in scaffold hopping), a more flexible approach is needed to select the optimal distance restraints between molecules.

In this work, we present a greedy algorithm to select (locally) optimal distance restraints for RBFE calculations with the linked dual-topology approach, which is also applicable to molecule pairs without a common core. In addition, the algorithm is extended to solve the same problem for multi-state RBFE methods such as enveloping distribution sampling (EDS) [41, 42] and multi-site $\lambda$-dynamics [43], resulting in a linked multi-topology approach. Finally, we analyze the sampling behavior and performance of the approach for the calculation of relative hydration free energies. The algorithm is implemented in a Python package (*https://github.com/rinikerlab/restraintmaker*), which can be used as a scripting library or with a GUI inside PyMOL [44].

## Theory

### End-state representations

**Fig. 2:**
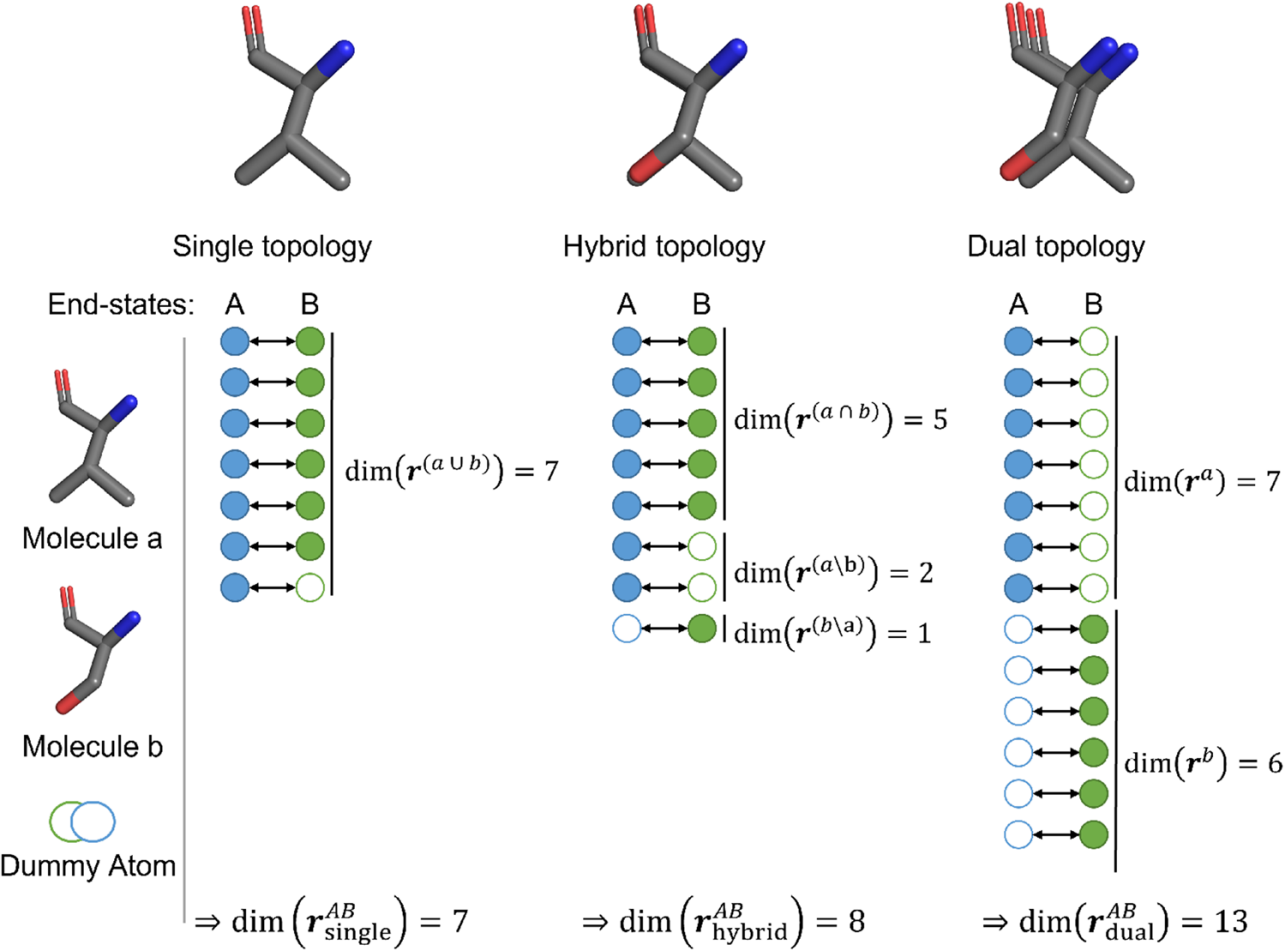
The three end-state representations can be illustrated using the coordinate mapping of molecules *a* and *b* in the end-states *A* and *B*. The smallest number of coordinates is required for the single-topology coordinate space ($dim(\mathbf{r}^{AB}_{\text {single}})$) as the coordinate set is formed from the union of all coordinates (left). If a coordinate is only used in one end-state, it becomes a non-interacting dummy atom in the other end-state. The hybrid-topology approach (middle) requires more coordinates for its coordinate space ($\mathbf{r}^{AB}_{\text {hybrid}}$), as the coordinates of differing atoms are represented separately. The largest coordinate space is required for the dual-topology approach. Here, the coordinate space ($dim(\mathbf{r}^{AB}_{\text {dual}})$) is the sum of both molecules.

In the following, we provide the categorization of the different system representation approaches (Fig. 2).

#### Single topology

The single-topology approach was first used by Jorgensen *et al.* [45] to calculate the relative hydration free energies of methanol and ethane. The approach was later termed “single-topology” by Pearlman et al. [29]. The end-states are represented by the union of the coordinates of the molecules, limiting the possible transformations that can be handled by this method. Usually, perturbations for a single topology approach include both atom types (i.e. van der Waals parameters and/or partial charges) and covalent terms [16, 17, 29, 30, 32, 45–50]. A single-topology approach in this definition is constructed as follows for two end-states *A* and *B* nvolving the two molecules *a* and *b* (Fig. 2),

$$\begin{aligned} dim(\mathbf{r}_{single}^{AB})&= dim(\mathbf{r}^{(a \cup b)}) \\ \mathbf{r}^{AB}_{\mathrm{single}}&= \{\mathbf{r}^{AB}_{1}, \mathbf{r}^{AB}_{2}, ..., \mathbf{r}^{AB}_{dim(\mathbf{r}_{single}^{AB})}\} \end{aligned}$$

where $dim(\mathbf{r}_{single}^{AB})$ is the total number of atomic coordinates of the end-states *A* and *B*, and $\mathbf{r}_{single}^{AB}$ the corresponding coordinate vector. Note that the unperturbed atoms of the environment (i.e., solvent and/or protein atoms), are not considered here for simplicity.

The single-topology approach has in principle the best sampling efficiency compared to other representations as it is constructed with the smallest number of coordinates and therefore the fewest degrees of freedom are perturbed [20, 30, 34, 47]. However, an issue arises when the molecules differ not only in the type of atoms but also in their number. To address this, a non-interacting “dummy” state can be assigned to the vanishing atom(s) [20, 30, 34, 47]. Different variants of dummy states are possible. Typically, only the non-bonded interactions are removed. However, it has been shown that also the covalent terms of “dummy” states can influence the free-energy calculations [34]. The construction of a single topology becomes increasingly challenging with more complex molecule transformations. For example, to realize complex transformations such as ring-size changes or ring opening/closing, special soft-bond terms have to be implemented [17].

#### Hybrid topology

The term hybrid single-dual topology was used by Jiang et al. [21] in 2019 to describe the combination of a single-topology core (common among the molecules) with dual-topology substituents (differing among the molecules). However, similar schemes were already used in previous studies (but called either single or dual topology) [15, 23, 37, 51–53]. A hybrid topology approach in this definition can be constructed as follows for two end-states *A* and *B* involving the two molecules *a* and *b* (Fig. 2),

$$\begin{aligned} dim(\mathbf{r}_{hybrid}^{AB})&= dim(\mathbf{r}^{(a \cap b)}) + dim(\mathbf{r}^{ (a \setminus b)}) + dim(\mathbf{r}^{~(b \setminus a)})\\ \mathbf{r}^{AB}_{\mathrm{hybrid}}&= \{\mathbf{r}^{~(a~\cap ~b)}_{1}, \mathbf{r}^{~(a~\cap ~b)}_{2}, ..., \mathbf{r}^{~(a~\cap ~b)}_{dim(\mathbf{r}^{~(a~\cap ~b)})}, \\&~~~\mathbf{r}^{~(a \setminus b)}_{1}, \mathbf{r}^{~(a \setminus b)}_{2}, ..., \mathbf{r}^{~(a \setminus b)}_{dim(\mathbf{r}^{~(a \setminus b)})}, \\&~~~\mathbf{r}^{~(b \setminus a)}_{1}, \mathbf{r}^{~(b \setminus a)}_{2}, ..., \mathbf{r}^{~(b \setminus a)}_{dim(\mathbf{r}^{~(b \setminus a)})}, \} \end{aligned}$$

Hybrid topology approaches aim at combining the advantages of single and dual topology, i.e., to keep the number of perturbed degrees of freedom minimal for sampling efficiency while facilitating more complex transformations.

#### Dual topology

In a dual topology, two fully separate sets of coordinates are used for the molecules. This approach was first introduced by Gao et al. [31], and termed later on “dual topology” by Pearlman et al. [29] The atoms of molecule *a*, which are fully interacting in end-state *A*, are transformed to the dummy state in end-state *B*, and vice versa. Importantly, the atoms of the two distinct molecules do not interact with each other and only share the same environment [15, 33]. Usually, in such dual topology approaches, only the non-bonded terms are perturbed [15, 19, 32, 54]. A dual-topology approach in this definition can be constructed as follows for the end-states *A* and *B* involving the two molecules *a* and *b* (Fig. 2),

$$\begin{aligned} dim(\mathbf{r}_{\mathrm{dual}}^{ AB})&= dim(\mathbf{r}^{ a}) + dim(\mathbf{r}^{ b})\\ \mathbf{r}^{AB}_{\mathrm{dual}}&= \{\mathbf{r}^{ a}_{1}, \mathbf{r}^{~a}_{2}, ..., \mathbf{r}^{~a}_{dim(\mathbf{r}^{ a})}, \mathbf{r}^{ b}_{1}, \mathbf{r}^{ b}_{2}, ..., \mathbf{r}^{ b}_{dim(\mathbf{r}^{ b})}\} \end{aligned}$$

The separated coordinates lead to a larger number of atoms in the system and thus, more degrees of freedom are perturbed, lowering the sampling efficiency compared to single-topology approaches. Three sub-variants of the dual-topology approach can be distinguished depending on how this issue is addressed in practice: (i) the linked variant with direct spatial restraints between the molecules to prevent them from drifting apart during the simulation [8, 15, 19, 55], (ii) the separated variant with restraining to the environment [33, 56], and (iii) the unconstrained variant [39, 57]. The linked dual topology is in principle the most efficient variant if the transformation is relatively simple (no changes in binding modes induced by reorientation or large conformational differences). The separated dual-topology approach is expected to be less efficient than the linked variant, but can handle these more challenging transformations [56]. A significant advantage of the dual-topology approach is the straightforward set-up of a system compared to the single and hybrid topologies, especially for more complex transformations or multiple end-states.

### Automated placement of distance restraints

To facilitate the set-up of RBFE calculations with the linked dual-topology approach, the selection of optimal distance restraints between the molecules needs to be automated. The proposed algorithm is based on classical graph algorithms. Its goal is to identify suitable placements for the distance restraints between two molecules $m_i$ and $m_j$. The following conditions are enforced:

1. $m_i$ and $m_j$ are pre-aligned to each other
2. Optimal placement of distance restraints requires that
  - the restrained atom pairs are maximally distant over the two molecules
  - the restrained atoms have a small distance to each other in the aligned structures
  - the restraints do not influence the conformational sampling of the molecules
3. For a user-defined number of required restraints $n_{\text {res}}$, it holds that $n_{\text {res}} \ll n_{m_i}$ and $~n_{res} \ll n_{m_j}$ , where $n_i$ and $n_j$ are the numbers of atoms of molecules $m_i$ and $m_j$, respectively

**Fig. 3:**
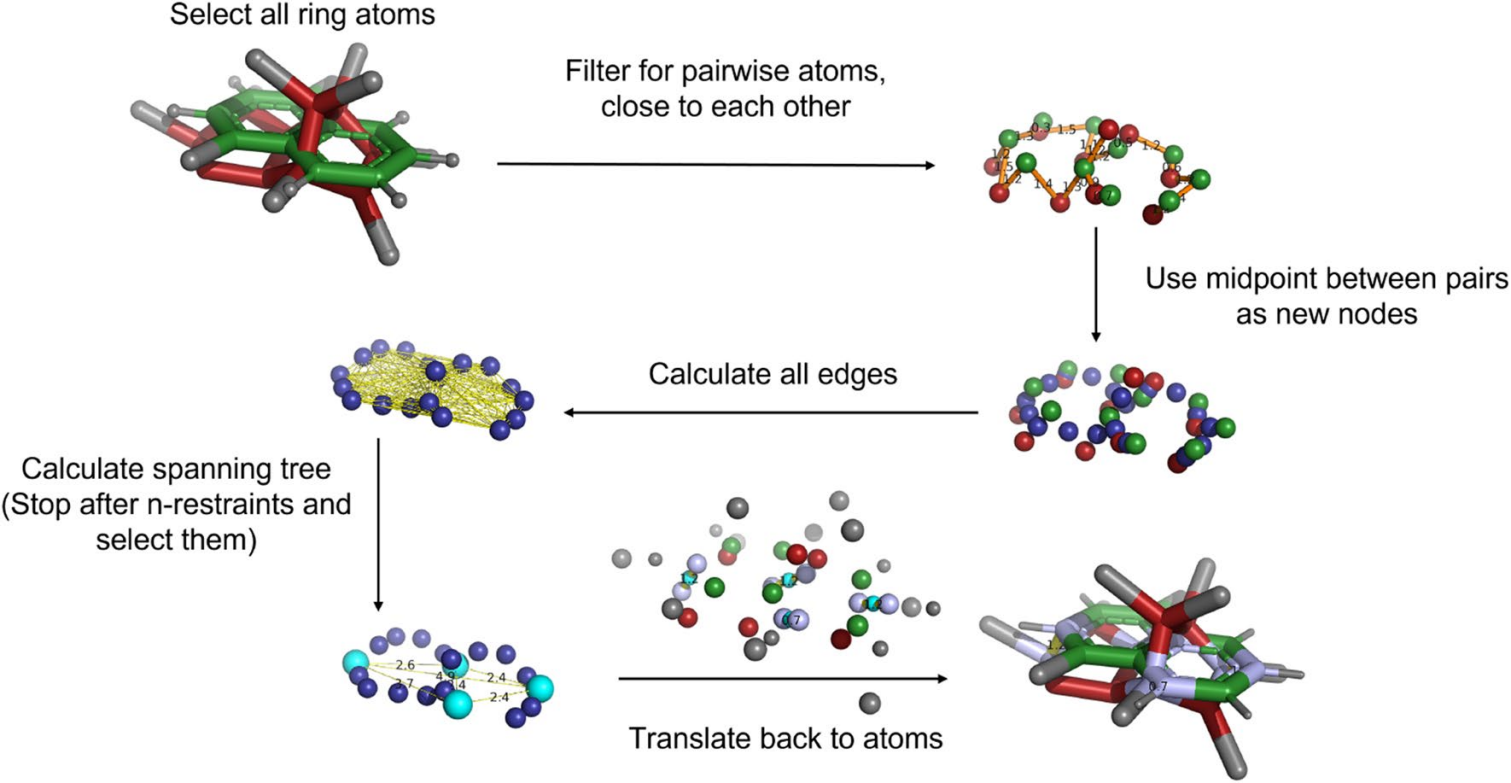
Schematic illustration of the algorithm steps to identify optimal placements for the distance restraints between a pair of molecules. The described algorithm uses a set of possible atoms (here these are the ring atoms). Next, possible restraints are filtered by the user-defined atom distance cutoff $d_\text {res}$. After this filtering step, the midpoints of the remaining possible restraints are calculated and used further as nodes of a graph. These nodes are connected by edges that have assigned weights, corresponding to the Euclidean distance of the midpoints. From this, a spanning tree is built with a min-max decision scheme. The construction of the spanning tree is stopped after $n_\text {res}$ iterations or if all nodes were connected. The result is a set of optimal restraints, $C_{\text {res,opt}}$, which will be translated back to an atom selection for further use in MD packages.

From these conditions follows that only atoms in relatively rigid regions of the molecules such as rings should be selected for the restraint search space. While restraining non-ring atoms might be favorable for achieving maximally distant distribution of the restrained atoms over the molecules, it is more likely to distort the conformational sampling of the molecules. The steps of the algorithm are shown schematically in Fig. 3 and explained in the following subsections.

#### Assigning distance restraints for a pair of molecules

*Translation into a graph problem*: The developed algorithm is based on a graph representation of the restraint search space. To solve the problem of selecting an optimal set of atom pairs to define the distance restraints, it needs to be translated into a graph *G* fulfilling,

1 $$\begin{aligned} G(N, E, \omega ), E \subseteq \{\{x,y\}\mid x,y\in N\;{\text {and}}\;x\ne y\}, \end{aligned}$$

where *N* is a set of nodes, *E* a set of edges, and $\omega$ a set of weights.

We consider a molecule pair consisting of the two molecules $m_i$ and $m_j$, with their sets of atoms $A_i$ and $A_j$, respectively. In a first step, existing algorithms such as e.g. implemented in the RDKit [58] or PyMol [44] can be used to align the two molecules onto each other. The alignment can for example be based on the maximum common substructure (MCS), or in the case of scaffold hopping the maximal overlap of the van der Waals volumes of the molecules. Next, the sets of possible atoms $A_i$ and $A_j$ for the distance restraints are reduced to the ring atoms of the respective molecules, $A^\text {ring}_i$ and $A^\text {ring}_j$. In addition, a user-defined cutoff distance $d_\text {res}$ between the pairs of atoms is introduced (here, $d_\text {res} = 0.1~\text {nm}$). Therefore, a possible distance restraint is a pair of atoms $(a^\text {ring}_i, a^\text {ring}_j)$ that fulfills the distance criterion $d(a^\text {ring}_i, a^\text {ring}_j) \le d_\text {res}$.

The possible distance restraints $C_\text {res}$ are used as nodes *N* to construct a fully connected graph *G*. Each individual restraint $c_\text {res}$ is represented by the midpoint of the two involved atoms. The undirected edges of *G* have as weight $\omega ^\text {dist}_{ji}$ the Euclidean distance between the midpoints of the two atom pairs $\omega ^{dist}_{ji}=d(c_{\text {res}_j},c_{\text {res}_i})$.

*Solving the graph problem*: From the generated graph, only a subset of restraints fulfills the conditions 1-3 listed above. To obtain a relevant subset of restraints, we use a min-max decision scheme inspired by the minimax theorem [59] to build a spanning tree (i.e. a subset of restraints, $C_\text {res,opt}$) within a greedy Prim-like approach [60].

The algorithm starts by picking the edge in the graph *G* with the largest weight $\omega ^\text {dist}_{ij}$ (distance), i.e. the two restraints whose midpoints are the furthest apart. After this initial selection of two restraints for $C_\text {res,opt}$, the weights of the edges in *G* are updated with the minimal distance of all $c_\text {res}$ in $C_\text {res,opt}$ to a respective node $n_i$. Subsequently, all edges and nodes are removed, which contain atoms that are already selected in $C_\text {res,opt}$. After the update of the edge weights, the restraint $c_\text {res}$ with maximal $\omega ^\text {dist}_{ji}$ is added to $C_\text {res,opt}$. This procedure is repeated until either $|C_\text {res,opt}| = n_\text {res}$ (in practice we expect a rather small number for $n_\text {res}$, typically $4< n_\text {res} < 10$) or all remaining nodes are connected.

*Back-mapping*: The selected subset of $n_\text {res}$ restraints, $C_\text {res,opt}$ is mapped back to the atoms in the molecules, such that the distance restraints can be written in a format readable by MD packages such as GROMOS [61] or GROMACS [62]. Additionally, a JSON [63] format is provided, allowing to import the results with any standardized JSON-Parser.

*Tie-breaking*: Due to non-perfect alignment and finite numerical precision, a tie between multiple restraints can occur, i.e. they have a very similar distance to the already selected restraints. This practical problem was solved by adding a tie-breaker that detects whether multiple high-priority restraints are within a range of 0.02 nm in a given iteration step and refines the decision result by applying a second criterion. For each candidate restraint in an iteration step, the distance to the center of geometry (COG) of all already selected restraints is calculated, and the restraint is chosen for which this distance is maximal.

#### Extension to multiple end-states

For multi-state methods such as EDS [41, 42], replica-exchange EDS (RE-EDS) [19, 64, 65], multi-site $\lambda$-Dynamics [43], or multi-state $\lambda$-LEUS [66], more than two molecules need to be restrained to each other. Based on our experience with RE-EDS, it is best to apply the distance restraints between multiple molecules in form of a ring, i.e. each molecule is restrained to two neighboring molecules [65]. This scheme is used in the following.

**Fig. 4:**
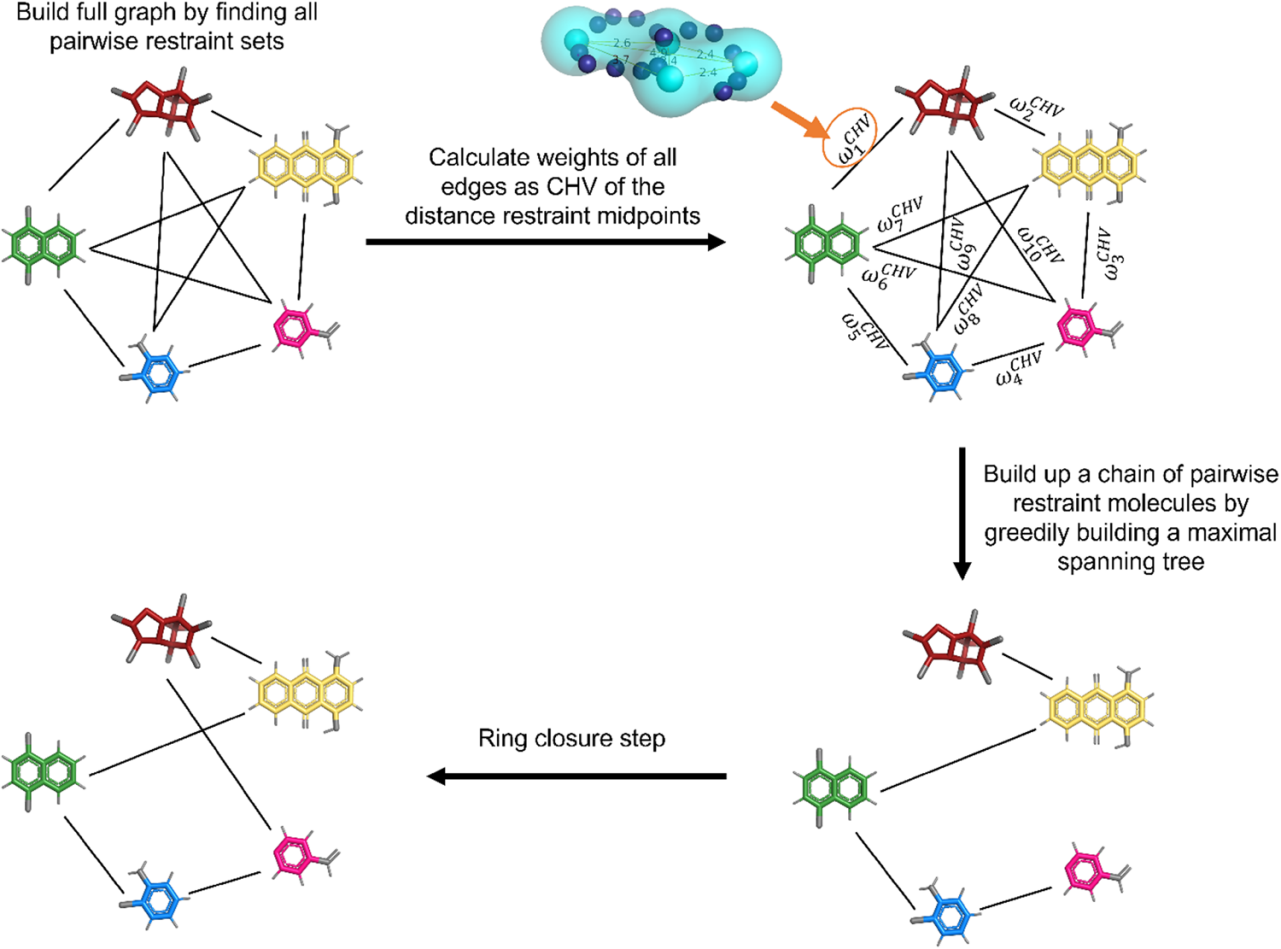
Schematic illustration of the algorithm steps to link multiple end-states by distance restraints for a multi-state RBFE calculation. The selection is carried out in four steps. (i) Optimal restraints are calculated for all possible molecule pairs, building up a fully connected graph. (ii) The weights $\omega ^\text {CHV}_i$ of the edges are calculated as the convex hull volume (CHV) formed by the selected restraints. (iii) A maximum spanning tree without branching is greedily constructed by selecting the edges with maximal weights. (iv) The ring is closed by connecting the ends of the chain.

In a first step, the pairwise greedy algorithm is used to calculate an optimal set of distance restraints between all possible molecule pairs, building up a fully connected graph (Fig. 4). The possible sets of restraints are subsequently compared to each other by calculating the convex hull around the coordinates of all the restraint midpoints. The convex hull volume (CHV) is then assigned to the edges of the fully connected graph as weight $\omega ^\text {CHV}$. The optimal chain of connected molecules is determined by applying another greedy algorithm, inspired by the Kruskal algorithm [67], which picks the edges with the largest CHVs without forming cycles or branches (Fig. 4). The chain is closed to a ring by tying the loose ends together. This last molecule pair may have a less good set of restraints.

### Free-energy methods

Two free-energy methods were tested with the linked dual-topology approach.

#### Thermodynamic integration

TI is a standard method to estimate free-energy differences [26], where a $\lambda$-dependent path between the two end-states *A* and *B* is sampled. The potential energy of the system is constructed as,

2 $$\begin{aligned} V(\mathbf{r}; \lambda ) = (1-\lambda ) ~ V_A(\mathbf{r}) + \lambda ~ V_B(\mathbf{r}) \end{aligned}$$

End-state *A* is obtained when $\lambda = 0$, and end-state *B* when $\lambda = 1$. In practice, simulations at discrete $\lambda$-points between 0 and 1 are performed, and the free-energy difference is obtained by numerical integration,

3 $$\begin{aligned} \Delta G_{BA} = \int ^{1}_{0} \left\langle \frac{\partial V(\lambda )}{\partial \lambda } \right\rangle _{\lambda } \,d\lambda \end{aligned}$$

#### Replica-exchange enveloping distribution sampling

RE-EDS [19, 64, 65] is a combination of Hamiltonian replica exchange [68, 69] and EDS [41, 42]. In EDS, a reference state $V_R$ is sampled, which combines *N* end-states as,

4 $$\begin{aligned} V_R\left( \mathbf{r};s,\mathbf{E}^R\right) = -\frac{1}{\beta s}\ln \left[ \sum \limits _{i=1}^N e^{-\beta s\left( V_i(\mathbf{r})-E_i^R\right) }\right] \end{aligned}$$

where *s* is the smoothness parameter, $E_i^R$ a set of energy offsets and $\beta =1/(k_B~T)$, where $k_B$ is the Boltzmann constant and *T* the absolute temperature. The force on a particle *k* can be calculated as [41, 42],

5 $$\begin{aligned} \mathbf{f}_k(t)=-\frac{\partial V_R(\mathbf{r}; s, \mathbf{E}^R)}{\partial \mathbf{r}_k} = \sum ^N_{i=1}\frac{e^{-\beta s(V_i(\mathbf{r}) -E_i^R)}}{\sum ^N_{j=1}{e^{-\beta s (V_j(\mathbf{r})-E_j^R)}}} \left( -\frac{\partial V_i(\mathbf{r})}{\partial \mathbf{r}_k} \right) \,. \end{aligned}$$

For high *s*-values (close to 1.0), the sampling of the reference state is dominated by the end-state with the lowest value of $V_i(\mathbf{r}) - E_i^R$, whereas for small *s* values (close to zero), all end-states contribute to the forces, resulting in the so-called “undersampling” [15].

The free-energy difference between a pair of end-states in the system is then calculated as,

6 $$\begin{aligned} \Delta G_{BA} = -\frac{1}{\beta }\ln \frac{\langle e^{-\beta (V_B-V_R)}\rangle _R}{\langle e^{-\beta (V_A-V_R}\rangle _R} \, . \end{aligned}$$

In practice, an optimal choice of *s* and $E_i^R$ is essential to sufficiently sample all end-states in an EDS simulation. RE-EDS overcomes the difficulty of choosing an optimal *s*-value by simulating multiple replicas with different *s*-values and performing replica exchanges between them [19, 64].

## Methods

### Validation of the restraint selection algorithm

To assess the performance of the greedy algorithm for selecting optimal distance restraints between two molecules, it was first tested on toy models. These contained 12 to 30 particles that were randomly distributed in space. The particles were randomly assigned to two entities representing two molecules. A selection of four restraints was performed with no pre-processing steps. Different algorithmic approaches were compared: the developed greedy algorithm, an averaged random selection (100 repetitions), and two brute-force approaches. One of the brute-force approaches maximizes the sum of the restraint midpoint distances between the selected restraints by considering all possible quadruples of restraints explicitly (BF-maxD). The other one maximizes the CHV around the selected restraints (BF-maxCHV), as done for chaining in multi-state systems. Each number of particles was sampled 20 times (using different particle coordinates each time) to provide an uncertainty estimate. The scripts for this validation are available in the example folder of the GitHub repository (*examples/publication/a_benchmark_algorithms*).

### Molecules with hydration free energies

**Fig. 5:**
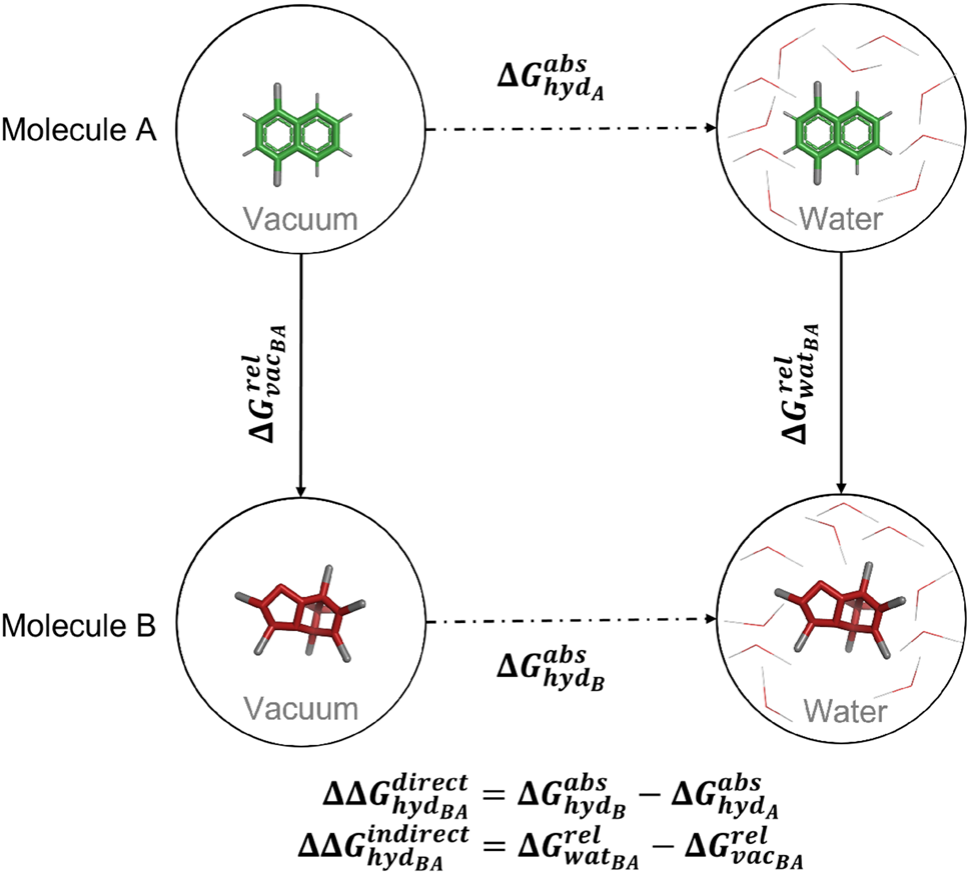
Thermodynamic cycle for the calculation of relative hydration free energies $\Delta \Delta G_{\text {hyd}_{AB}}$. The direct way to obtain $\Delta \Delta G_{\text {hyd}_{AB}}$ employs two absolute free-energy calculations giving $\Delta G^\text {abs}_{\text {hyd}_A}$ and $\Delta G^\text {abs}_{\text {hyd}_B}$. The indirect way uses two alchemical or relative free-energy calculations giving $\Delta G^\text {rel}_{\text {vac}_{AB}}$ and $\Delta G^\text {rel}_{\text {wat}_{AB}}$.

The algorithm was applied to the calculation of relative hydration free energies $\Delta \Delta G_{\text {hyd}}$ (Fig. 5). A set of 16 molecules with experimentally available hydration free energies [70–75] was selected (Table S1 in the Supporting Information). The topologies for these molecules were taken from the ATB server [76]. The selected molecules are small and possess a ring core. The corresponding pairwise transformations are nevertheless relatively complex, and involve R-group and ring-size changes as well as scaffold hopping-type transformations (e.g. benzene to cyclohexane).

**Fig. 6:**
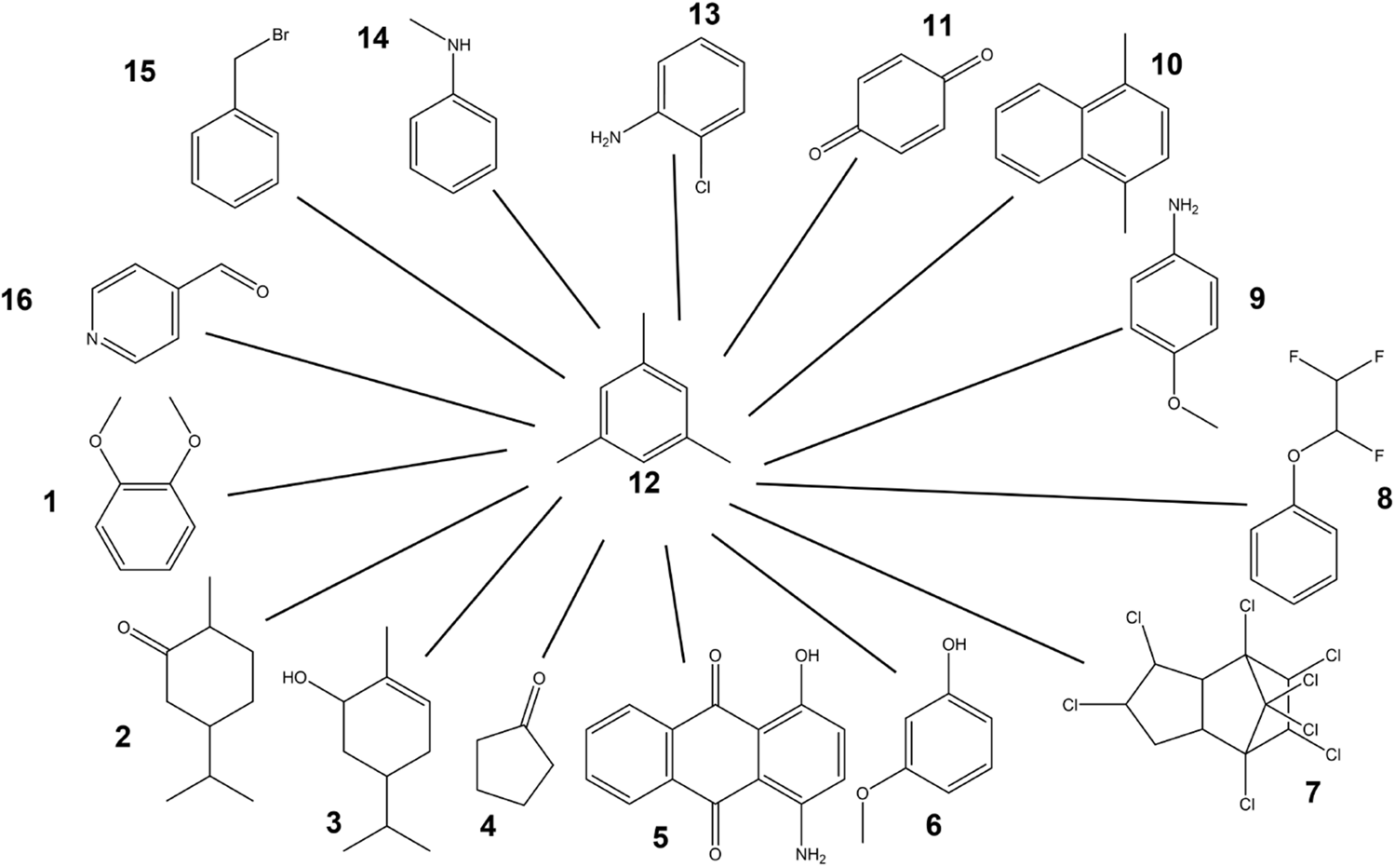
Set of 16 molecules with experimental hydration free energies available [70–76]. The black lines indicate the pairs of molecules for which TI calculations were performed. RestraintMaker was used to select pairwise distance restraints between the central molecule and all others (Fig. S1 in the Supporting Information).

Pairwise TI calculations were carried out with a linked dual topology approach for the 16 molecules in a star-like scheme with molecule **12** as center, resulting in 15 relative hydration free energies (Fig. 6).

**Fig. 7:**
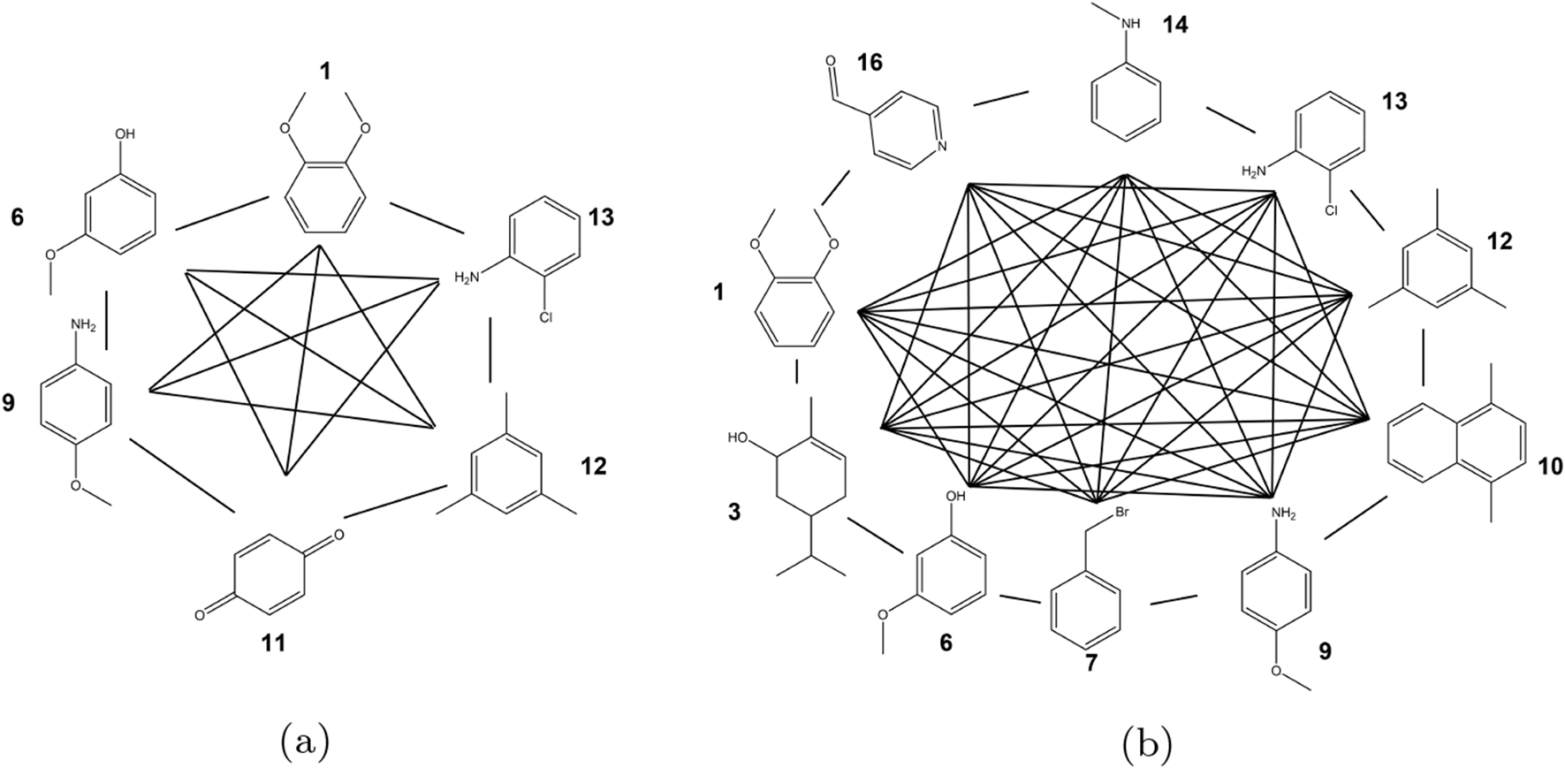
Subsets A and B used for the RE-EDS simulations. The black lines indicate the relative hydration free energies that can be extracted. (a): Subset A consists of six molecules with the same benzene core and R-group changes. The distance restraints selected by RestraintMaker are shown in Figs. S2 (TI) and S3 (RE-EDS) in the Supporting Information. (b): Subset B consists of ten molecules without a common core, involving ring-flexibility changes. The distance restraints selected by RestraintMaker are shown in Fig. S4 in the Supporting Information.

For RE-EDS, two subsets of the 16 molecules were generated. The first subset A contains six molecules with the same benzene core and R-group changes (Fig. 7a). For this set A, all relative hydration energies were also estimated with pairwise TI calculations. The second subset B consists of ten molecules without a common core, involving more complex transformations such as ring-flexibility changes (Fig. 7b).

### Simulation details

All simulations were carried out using the MD software package GROMOS [61] version 1.5.0 (freely available on *http://www.gromos.net*) [65], the Python RE-EDS pipeline (*https://github.com/rinikerlab/reeds*) and PyGromosTools [77] (*https://github.com/rinikerlab/PyGromosTools*).

In order to compare our results with the absolute hydration free energies reported in the ATB server [76], the same simulation setup was used. The simple point-charge (SPC) model [78] was employed for water. A single cutoff radius of 1.2 nm was used for the calculation of the non-bonded interactions. The integration time step was set to 2 fs and the pairlist was updated every five steps. Long-range nonbonded interactions were calculated using a reaction-field correction [79] with $$\varepsilon _{\text {rf}}=1$$ for the simulations in vacuum and $$\varepsilon _{\text {rf}}=61$$ for the simulations in water [80]. The force constant for the distance restraints was set to 5000 kJ/(mol$$\cdot $$nm$$^2)$$.

#### Thermodynamic integration

The topologies and coordinate files of the single states were obtained from the ATB server [76] and merged to pairs using PyGromosTools [77]. The coordinates of the different molecules were aligned to each other using the common molecular skeleton of the molecules (only rings), with the *align* function in RDKit [58]. After the alignment, RestraintMaker was used to place four restraints with $d_\text {res} = 0.1$ nm (Figs. S1 and S2 in the Supporting Information). The computational boxes for the simulations in water were generated with the GROMOS++ [81] program *sim_box* using a minimal solute-to-wall distance of 0.8 nm, and relaxed by energy minimization. The scripts can be found in the example folder on Github (https://github.com/rinikerlab/restraintmaker/tree/main/examples/publication/b_ATB_solvationFreeEnergies). The TI calculations were carried out with at 21 evenly spaced $\lambda$-points between 0 and 1, for both the molecules in water and in vacuum. Each $\lambda$-point was equilibrated for 1 ns, followed by a production run of 5 ns. The free-energy differences were calculated using the Simpson integration implemented in the SciPy library [82].

#### RE-EDS

The merged topologies for the RE-EDS calculations were prepared using PyGromosTools [77], and the simulations were set-up using the RE-EDS pipeline [65]. The individual steps of the pipeline are described in detail in Ref. [65]. The coordinates of the molecules were aligned to reference molecule **1** (Fig. 6). The RDKit [58] was used to determine the pairwise MCSs (using the molecular skeleton of the rings only) and align the molecules based on it. For some molecules, manual modifications were applied to ensure an optimal overlap of the ring atoms and substituents. The corresponding script is available in the example folder on GitHub (https://github.com/rinikerlab/restraintmaker/blob/main/examples/publication/b_ATB_solvationFreeEnergies/sets/multistate/prepare_distance_restraints.py). The RestraintMaker was then used to select the distance restraints to connect the molecules in a chain using analogous parameters as for the pairwise restraints for TI (Figs. S3 and S4 in the Supporing Information).

For subset A (Fig. 7a), six EDS simulations of 2 ns length were carried out with $s=1.0$ in vacuum/water to generate optimized configurations for the starting state mixing (SSM) [65]. Each of the six simulations was biased towards one of the end-states by setting the energy offset of that end-state to 500 kJ mol$^{-1}$ and the energy offsets of the other end-states in the same simulation to – 500 kJ mol$^{-1}$. Subsequently, 21 EDS simulations were carried out for 0.2 ns with *s*-values distributed logarithmically between 1 and $10^{-5}$ to determine the lower bound for the RE-EDS simulations (0.00316 in vacuum and 0.001 in water). Next, the energy offsets were estimated from a 0.8 ns RE-EDS simulation, with 12 replicas in vacuum, and 14 replicas in water. In vacuum, one *s*-optimization step of 0.5 ns length adding four replicas was sufficient to achieve frequent round trips and good sampling of all end-states. In water, three *s*-optimization steps of 0.5 ns, 1.0 ns and 1.5 ns, respectively, were carried out to achieve frequent round trips. At each step, five replicas were added. Additionally, in water, the energy offsets were rebalanced over three 0.5 ns simulations to optimize the sampling of all end-states. Energy-offset rebalancing was not necessary in vacuum as all end-states were already sampled well after the *s*-optimization. The production run was 0.5 ns long in vacuum and 1.0 ns in water.

For subset B, ten EDS simulations of 2 ns were performed in vacuum/water to generate optimized configurations analogously to set A. The determination of the lower bounds was carried out as above (0.00178 in vacuum and 0.001 in water). For the energy offset estimation, a 0.8 ns RE-EDS simulation was used again, with 17 replicas in vacuum and 18 replicas in water. In vacuum, one *s*-optimization step of 0.5 ns length adding four replicas was also sufficient. In water, five *s*-optimization steps of 0.5 ns, 1.0 ns, 1.5 ns, 1.5 ns, and 1.5 ns, respectively, were carried out to achieve frequent round trips. At each step, five replicas were added. In water, the energy offsets were rebalanced over five 0.5 ns simulations. The production run was 1.0 ns long in vacuum and 5.0 ns in water.

### Analysis

The analysis of the simulations was carried using GROMOS++ [81] and PyGromosTools [77]. In addition, the following Python packages were employed for the statistical analysis and plotting: Pandas [83], Matplotlib [84], NumPy [85], SciPy [82], mpmath [86], and Jupyter notebooks [87].

## Results and discussions

### Validation of the restraint selection algorithm

As a greedy algorithm, the approach in RestraintMaker might lead to sub-optimal solutions. To test this, the performance of the algorithm was compared with that of two brute-force approaches. One brute-force approach, BF-maxD, maximizes the distance of all selected restraint midpoints to each other, whereas the second brute-force approach, BF-maxCHV, maximises instead the CHV spanned by the selected restraint midpoints. Toy systems consisting of two strongly overlapping particle clouds were constructed, for which four restraints should be selected. The systems varied in the number of randomly placed particles. Each particle mimics an atom of a hypothetical molecule that might be selected to be restrained.

**Fig. 8:**
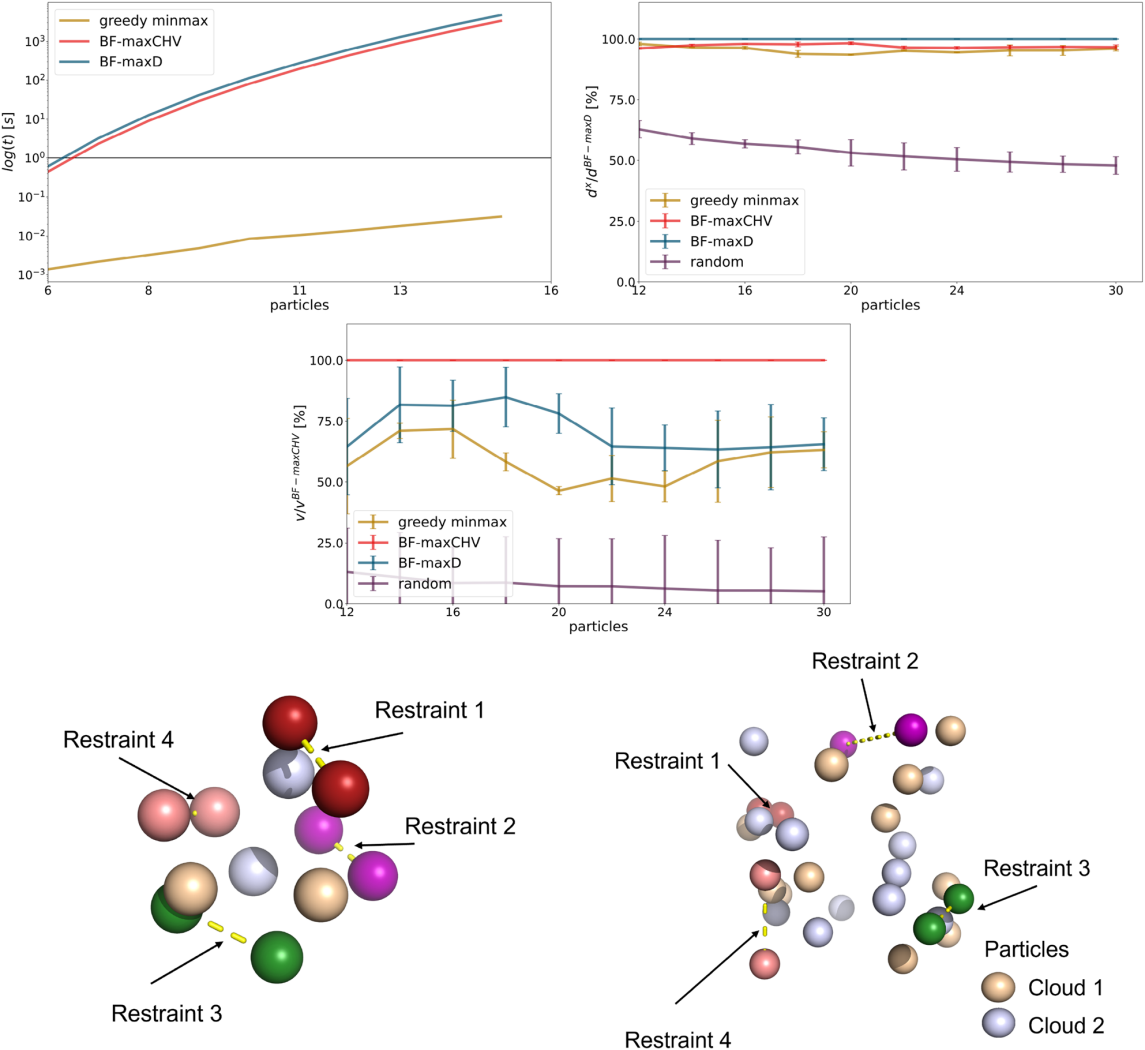
Comparison of algorithms to select distance restraints on toy systems. (Top left): Time complexity as a function of the number of particles in the system for the brute-force approaches BF-maxCHV (red) and BF-maxD (blue), and the greedy algorithm (yellow). (Top right):): Distance metric as a function of the number of particles in the system for the brute-force approaches (red and blue), the greedy algorithm (yellow), and random selection with 100 trials (purple). (Middle): CHV as a function of the number of particles in the system. (d and e): Final distance restraints selected by the greedy algorithm for 12 (bottom left) and 30 (bottom right) particles. The restrained atoms are colored in green, red, pink and rose, and connected by yellow dashed lines. The two particle clouds are colored in wheat and light blue.

The advantage of the greedy algorithm is evident when considering the time complexity as the brute-force approaches scale with $\mathcal O(N^4)$ (where *N* is the number of atoms), making them unusable for larger molecules (Fig. 8a). For selecting four restraints in the 20 particles toy system, the BF-maxCHV requires 75 s (single core), and 3325 s for 30 particles. In comparison, the greedy algorithm requires only 0.031 s for the 30 particles.

Comparison of the sum of all distances between restraint midpoints shows that BF-maxD (optimizing for the maximal distance between all midpoints of the selected restraints) yields the best results with the largest distances (blue line in Fig. 8b). The second brute-force algorithm BF-maxCHV (optimizing for the CHV defined by the restraint midpoints) and our greedy algorithm give comparable results to BF-maxD. All three approaches are very good at maximizing the distance between the selected restraints. Random selection with 100 trials (negative control), on the other hand, performs significantly worse. Second, we compared the approaches based on the CHV generated by the selected restraints (Fig. 8c). Here, the BF-maxCHV approach yields the best results as expected, while BF-maxD and the greedy algorithm perform similar to each other. The difference in CHV between the latter two approaches and BF-maxCHV increases with increasing number of particles in the toy system. This may be due to the growing number of possible choices, or due to the fact that the distance metric used in BF-maxD and the greedy algorithms is suboptimal. All approaches clearly outperform random selection. The greedy algorithm in RestraintMaker can thus be seen as a trade-off between optimizing a metric (distance or CHV) and limiting the required computing time. It is the fastest algorithm among the ones tested and yields comparable results to the brute-force approaches.

### Calculation of relative hydration free energies

#### Thermodynamic integration

**Fig. 9:**
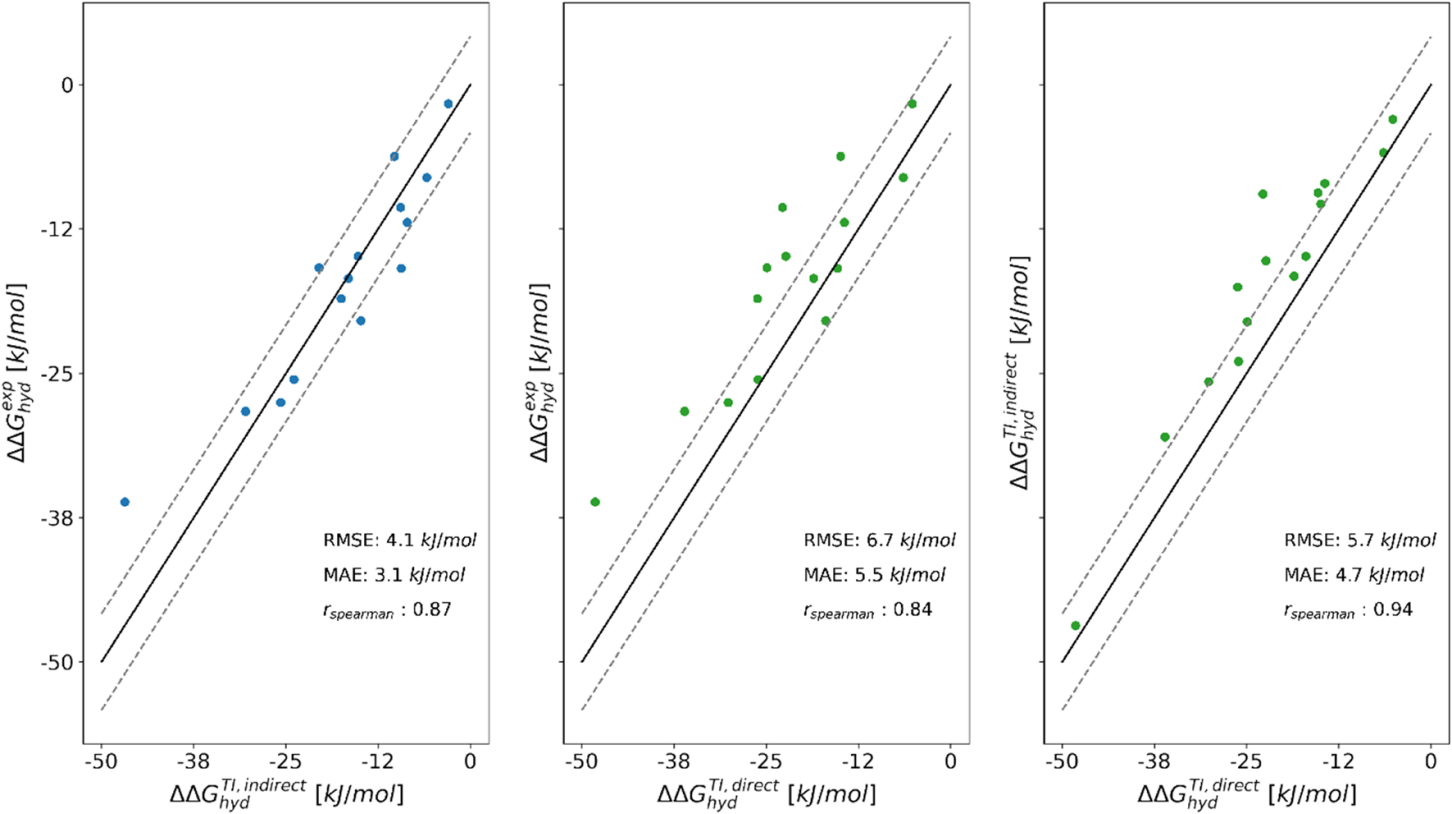
Comparison of the relative hydration free energies $\Delta \Delta G_{hyd}$ (with molecule **12** as reference) for the 16 small molecules between experiment (exp), the pairwise relative free-energy calculations with TI and linked dual topology (TI, indirect), and the absolute free-energy calculations with TI taken from the ATB server [76] (TI, direct). The numerical values are given in Table S2 in the Supporting Information.

To assess the quality of the selected distance restraints, the greedy algorithm in RestraintMaker was tested with a set of 16 small molecules with experimentally available hydration free energies. First, the relative hydration free energies were calculated between molecule **12** and the 15 other molecules using TI (Fig. 6). The resulting $\Delta \Delta G_\text {hyd}^\text {TI,indirect}$ agree very well with the experimental values [70–75], with a root-mean-square error (RMSE) of 4.1 kJ/mol, a mean absolute error (MAE) of 3.1 kJ/mol (Fig. 9, left) and a Spearman correlation coefficient $r_{\text {Spearman}}$ of 0.87. The numerical values are reported in Table S2 in the Supporting Information, and the corresponding $\langle \partial V(\lambda )/\partial \lambda \rangle$ curves in water and vacuum in Figs. S5 and S6.

For comparison, $\Delta \Delta G_{hyd}^\text {TI,direct}$ values were derived from the calculated absolute hydration free energies reported in the ATB server [76], which were carried out with TI using the same topologies. The $\Delta \Delta G_{hyd}^{TI,direct}$ values deviate slightly more from experiment with an RMSE of 6.7 kJ/mol, a MAE of 5.5 kJ/mol and a $r_{\text {Spearman}}$ of 0.84 (Fig. 9, center). Generally, the results of the direct [76] and indirect TI calculations agree well with each other (Fig. 9, right). Note that for the molecule pair **5**–**12**, a similarly large deviation from experiment is observed in both types of TI calculations (10.7 kJ/mol and 12.1 kJ/mol, respectively), suggesting either a force-field deficiency or a problematic experimental value.

**Fig. 10:**
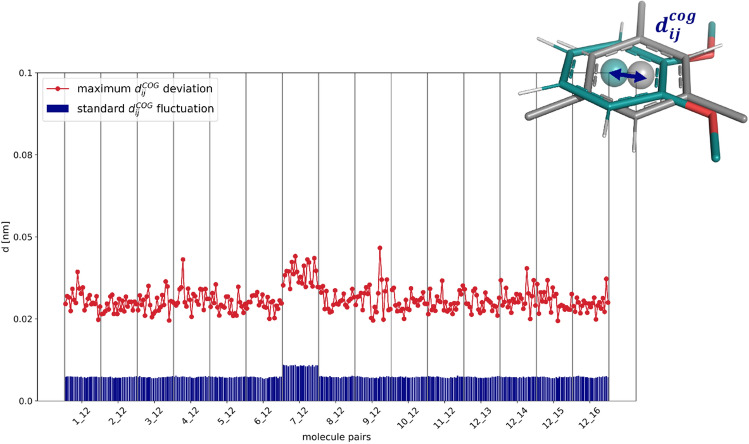
Standard deviation of the distance distribution (blue) and maximum distance (red) between the COGs of the central rings of the molecule pairs in the TI simulations in water. The COG was calculated for the restrained atoms in the rings. The horizontal axis shows for each molecule pair the different $\lambda$-windows between 0 and 1.

It is crucial for the linked dual-topology approach that the applied distance restraints do not distort the conformational sampling of the molecules. For this, the relative translational motion of the two aligned molecules was analyzed for each $$\lambda $$-window and molecule pair by calculating the fluctuation of the distance between the COGs of the restrained atoms in the central rings of the two molecules. Figure 10 shows both the standard deviation and the maximum observed distance between the two COGs. The standard deviation is close to zero for all pairs, indicating that the two cores overlap well given the chosen restraints. The maximum distances are around 0.03 nm. For the pair **7**–**12**, the distances are slightly higher, which results from the fact that molecule **7** is a bridged bicycle. The force constant of 5000 kJ/(mol$$\cdot $$nm$$^2)$$ for the distance restraints is found to represent a good compromise to ensure a tight overlap of the molecules without significantly perturbing their conformations. Note that the range of reasonable force constants is rather large and only for extremely high values (i.e. 50’000 kJ/(mol$$\cdot $$nm$$^2)$$ or larger), does the restraining affect the free-energy results.

**Fig. 11:**
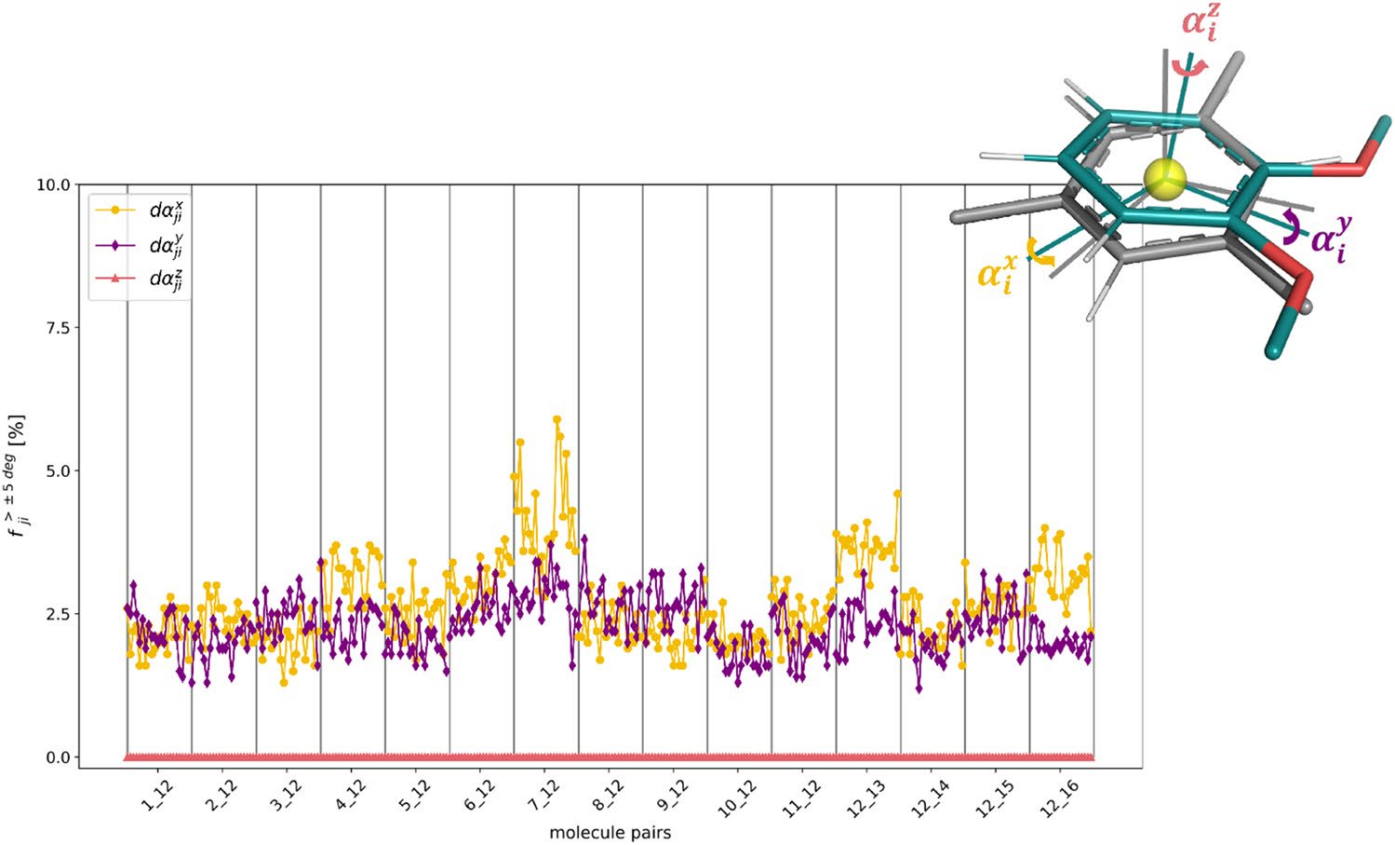
Fraction *f* of frames in the TI simulations in water, in which the relative rotation around the *x*-axis (yellow), *y*-axis (purple), and *z*-axis (red) of the central rings of the molecule pair exceeds $5^{\circ }$. The horizontal axis shows for each molecule pair the different $\lambda$-windows between 0 and 1.

A similar analysis was carried out for the relative rotational motions of the molecules, considering the restrained atoms in the molecule pairs (Fig. 11). In terms of the three Euler angles, a maximum relative rotation of $6.3^\circ$ was observed, which is reasonably small for one dimension. The largest fluctuation was again observed for the pair **7**–**12**. The rotation around the *z*-axis shows significantly smaller deviations compared to the other dimensions, because the two molecules need to rotate against each other in plane. In contrast, the rotations around the *x* and *y*-axis correspond to a relative tilt of the molecules, which is easier to realize.

**Fig. 12:**
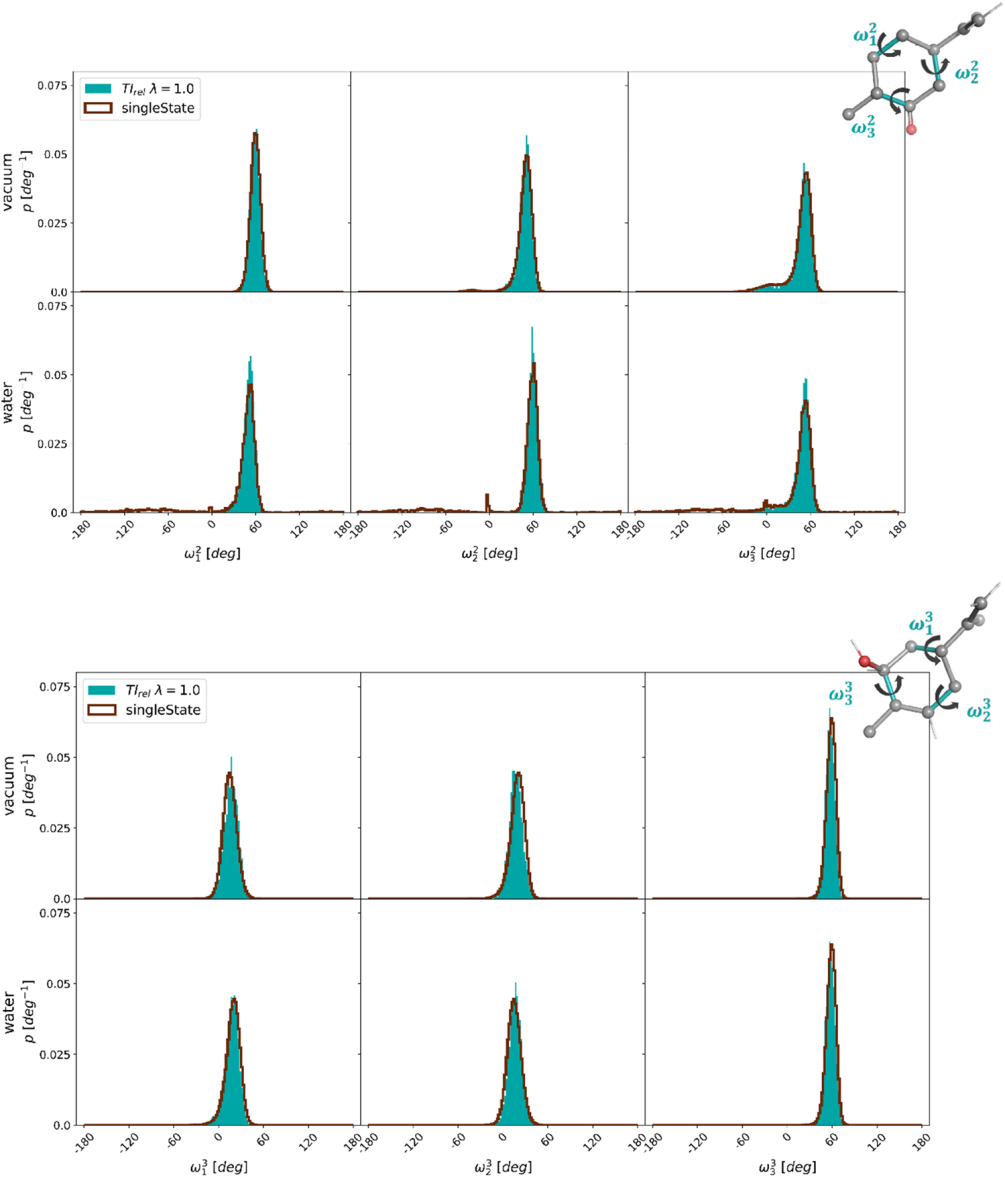
Comparison of the normalized torsional angle distributions of the three pseudo torsional angles of the aliphatic ring of molecules **2** (top) and **3** (bottom) in the TI calculation at $\lambda =1.0$ (filled) and in plain MD simulations (dark red line) in vacuum (top) and in water (bottom).

While most of the molecule pairs in Fig. 6 have the same central benzene core, the transformations from molecule **12** to molecules **2** and **3** involve the change from benzene to cyclohexane or cyclohexene, respectively. To assess whether the applied distance restraints affect the conformational sampling of the aliphatic ring, the distributions of the three pseudo torsional angles (Pickett and Strauss coordinates [88]) were monitored in the simulation at $\lambda =1.0$, and compared to plain MD simulations of molecules **2** and **3** in vacuum and in water (Fig. 12). In both cases, the distributions showed nearly perfect overlap, indicating that the sampling is not affected by the distance restraints in the linked dual topology.

A similar analysis of the torsional angle distributions was also carried out for the substituents of molecules **1**, **6** and **9** (Fig. S8 in the Supporting Information). Again, no major differences are observed between $\lambda =1.0$ and the plain simulations.

#### Multi-state simulations with RE-EDS

Multi-state simulations were performed with RE-EDS for the two subsets A and B (Fig. 7). The distance restraints were selected with the developed algorithm using the CHV approach, connecting always one molecule with two others.

**Fig. 13:**
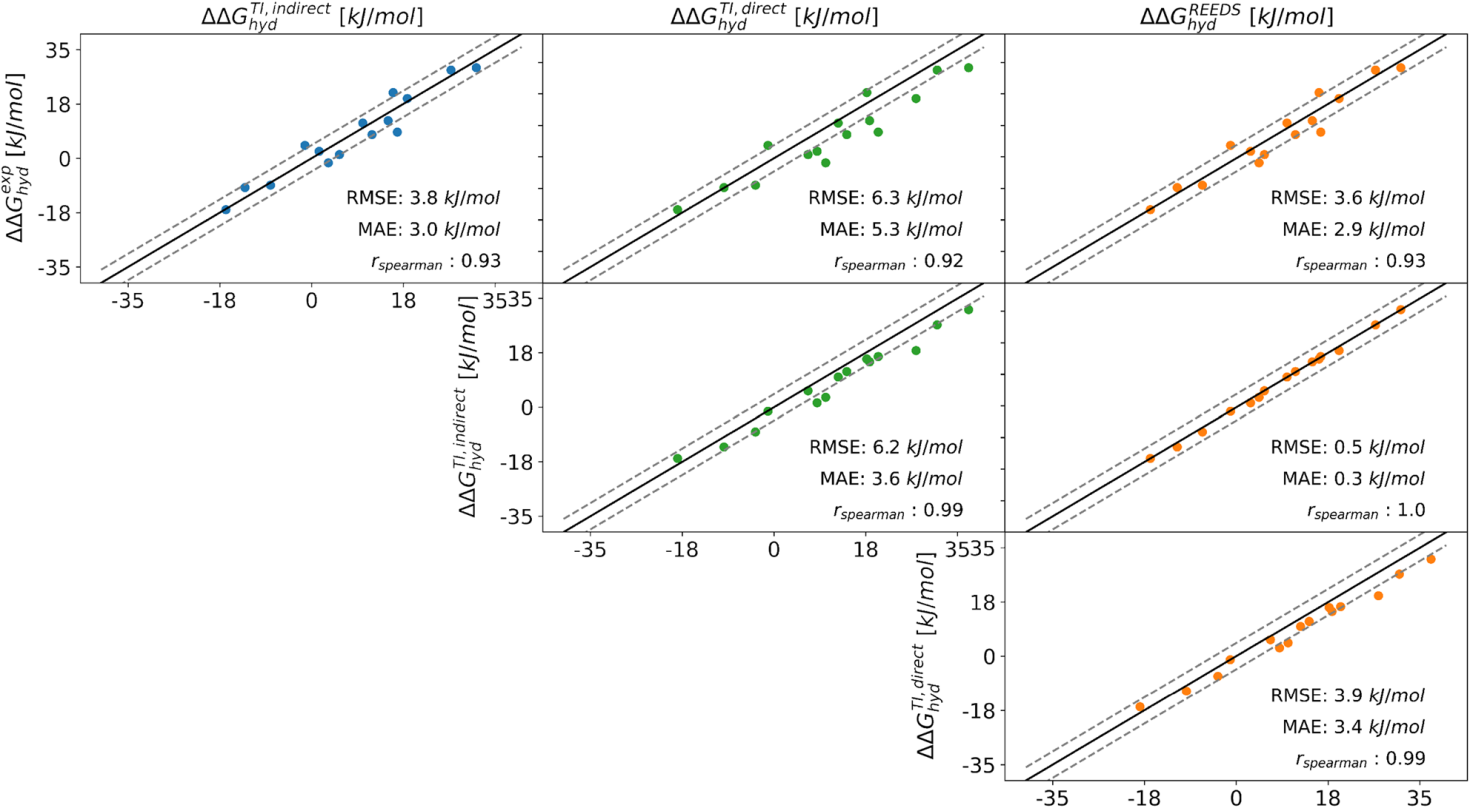
Comparison of the relative hydration free energies $\Delta \Delta G_{hyd}$ for the six molecules in subset A between experiment (exp), the multi-state relative free-energy calculations with RE-EDS and linked dual topology (RE-EDS, indirect), the pairwise relative calculations with TI and linked dual topology (TI, indirect), and the absolute free-energy calculations with TI taken from the ATB server [76] (TI, direct). The corresponding statistical metrics are reported in Table 1. The numerical values are given in Table S3 in the Supporting Information.

Subset A consists of six molecules. To be able to directly compare with the $\Delta \Delta G_{hyd}$ values from RE-EDS, TI calculations were also performed for the 15 pairwise transformations in Fig. 7a. The results using RE-EDS and TI agree very well with each other as well as with experiment (Fig. 13), with an RMSE of 3.8 kJ/mol and a MAE of 3.0 kJ/mol for TI, and an RMSE of 3.6 kJ/mol and a MAE of 2.9 kJ/mol for RE-EDS. The numerical values are reported in Table S3 in the Supporting Information. For both methods, the Spearman correlation coefficient is 0.93, indicating high correlation with experiment. The highest deviations from experiment are observed for the molecule pair **6**–**11** for both relative methods with deviations of 8.3 kJ/mol and 7.5 kJ/mol, respectively. For comparison, $\Delta \Delta G_\text {hyd}^\text {TI,direct}$ were derived from the absolute hydration free energies reported in the ATB server [76], giving an RMSE of 6.2 kJ/mol and a MAE of 5.3 kJ/mol. The Spearman correlation coefficient is almost identical with a value of 0.92. In this case, the highest deviations from experiment are observed for the molecule pairs **1**–**11** and **6**–**11**, with deviations of 11.1 kJ/mol and 11.3 kJ/mol, respectively.

These results highlight the advantage of using RE-EDS for relative free-energy calculations (Table 1). The accuracy of the results is similar for TI and RE-EDS, but RE-EDS is considerably more efficient with a total simulation time (pre-processing plus production) of about 260 ns. Using TI, a total simulation time (equilibration plus production) of about 3800 ns was required for calculating all 15 pairs in subset A. This time can of course be reduced to about 1265 ns when calculating only the minimal number of five pairs. In addition, the length of the production runs could be reduced from 5 to 3 ns for many pairs, without affecting convergence. However, even with these reductions, the total required simulation time with TI is still about three times longer than with RE-EDS. The convergence for both methods is shown in Figs. S7 and S9 in the Supporting Information.

**Table 1:** Comparison of statistical metrics (RMSE, MAE, and Spearman correlation coefficient relative to experiment) as well as the accumulated simulation time between the different free-energy methods. The simulation time is split into preparation (pre-processing, equilibration) and production run. For relative TI calculations, the time for the minimal number of pairs is reported (five for subset A, and nine for subset B). Calculated absolute hydration free-energies were taken from the ATB server [76] to calculate the relative hydration free energies $\Delta \Delta G_{hyd}^{direct}$. The uncertainty for the RMSE values was estimated by a 100 times iterated bootstrap approach. The corresponding correlations are shown graphically in Figs. 9, 13 and 14.

	Subset A			Subset B		
	$\Delta \Delta G^{direct}_{hyd}$	$\Delta \Delta G^{indirect}_{hyd}$		$\Delta \Delta G^{direct}_{hyd}$	$\Delta \Delta G^{indirect}_{hyd}$	
	TI [76]	TI	RE-EDS	TI [76]	TI	RE-EDS
RMSE $[\text {kJ/mol}]$	6.2 ± 0.3	3.7 ± 0.1	3.6 ± 0.3	5.8 ± 0.1	3.4 ± 0.1	3.3 ± 0.1
MAE $[\text {kJ/mol}]$	5.3 ± 3.2	3.0 ± 2.1	3.0 ± 2.2	4.8 ± 3.3	2.8 ± 2.0	2.7 ± 1.8
$\text {r}_{\text {Spearman}}$	0.92	0.93	0.93	0.93	0.96	0.96
$\text {t}_{\text {preparation}}~[\text {ns}]$	−	215	222	−	378	418
$\text {t}_{\text {production}}~[\text {ns}]$	$42-102$	1050	36	$70-170$	1890	212

As for the pairwise TI calculations, the effect of the applied distance restraints on the conformational sampling in the RE-EDS simulations was evaluated. Both the translational and rotational fluctuations of the COGs of the central rings are comparable to those observed in the TI calculations (Figs. S10 and S11 in the Supporting Information). Similarly, the reweighted torsional angle distributions of the substituents of molecules **1**, **6** and **9** agree well with those from plain MD simulations (Fig. S12 in the Supporting Information).

**Fig. 14:**
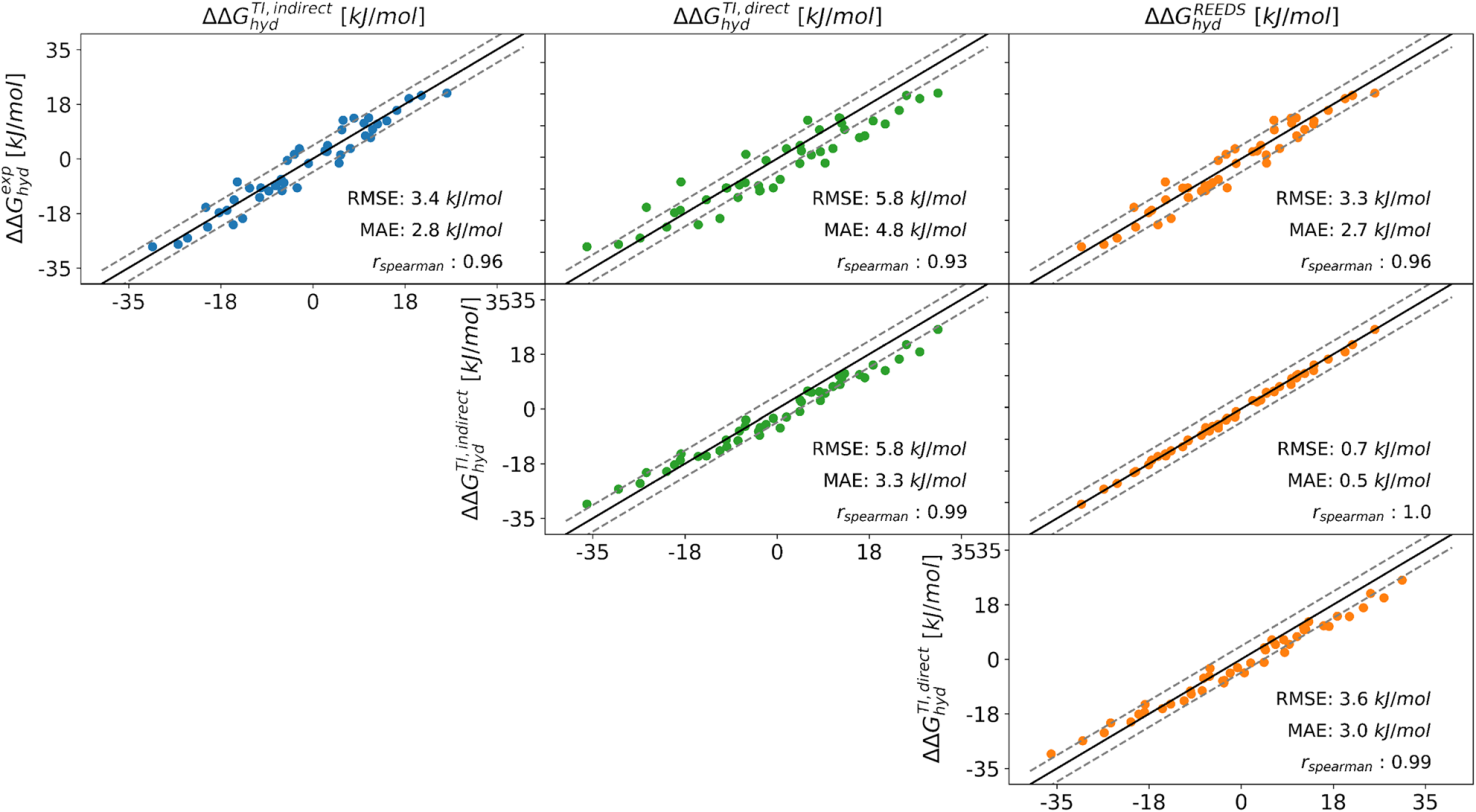
Comparison of the relative hydration free energies $\Delta \Delta G_\text {hyd}$ for the six molecules in subset B between experiment (exp), the multi-state relative free-energy calculations with RE-EDS and linked dual topology (RE-EDS, indirect), the pairwise relative calculations with TI and linked dual topology (TI, indirect), and the absolute free-energy calculations with TI taken from the ATB server [76] (TI, direct).

RE-EDS simulations were also performed with the second subset B, consisting of ten molecules (Fig. 7b). For comparison, the results calculated from the minimal number of nine molecule pairs were taken from the previous TI calculations (i.e., with molecule **12** at the center). The results were used to estimate the remaining 36 $\Delta \Delta G_\text {hyd}^\text {TI,indirect}$ values. As for subset A, the results with RE-EDS and TI agree very well with each other and also with experiment (Fig. 14), with an RMSE of 3.4 kJ/mol and a MAE of 2.8 kJ/mol for TI, and an RMSE of 3.3 kJ/mol and a MAE of 2.7 kJ/mol for RE-EDS. The Spearman correlation coefficient with experiment is 0.96 for both methods. The highest deviations from experiment are observed for the molecule pairs **14**–**9**, **14**–**10**, **15** - **10**, and **16** - **14** for both methods, with absolute deviations between 6.1 and 6.9 kJ/mol. For comparison, the $\Delta \Delta G_\text {hyd}^\text {TI,direct}$ values were again derived from the absolute hydration free energies reported in the ATB server [76], giving an RMSE of 5.8 kJ/mol and a MAE of 4.8 kJ/mol. The Spearman correlation coefficient is almost identical with a value of 0.93. In this case, the highest deviations from experiment are observed for the molecule pairs **10**–**1**, **10**–**3**, **10**–**6**, **14**–**10**, **14** - **12**, and **15**–**10**, with absolute deviations between 9.1 and 10.7 kJ/mol.

For the RE-EDS simulations, a total simulation time of about 630 ns was used for subset B, yielding all 45 $\Delta \Delta G_{hyd}$ values simultaneously (Table 1). When simulating the minimal number of nine molecule pairs with TI, the total simulation time was about 2000 ns (or about 1100 ns with 3 ns production runs). The convergence for both methods is shown in Figs. S7 and S13 in the Supporting Information.

**Fig. 15:**
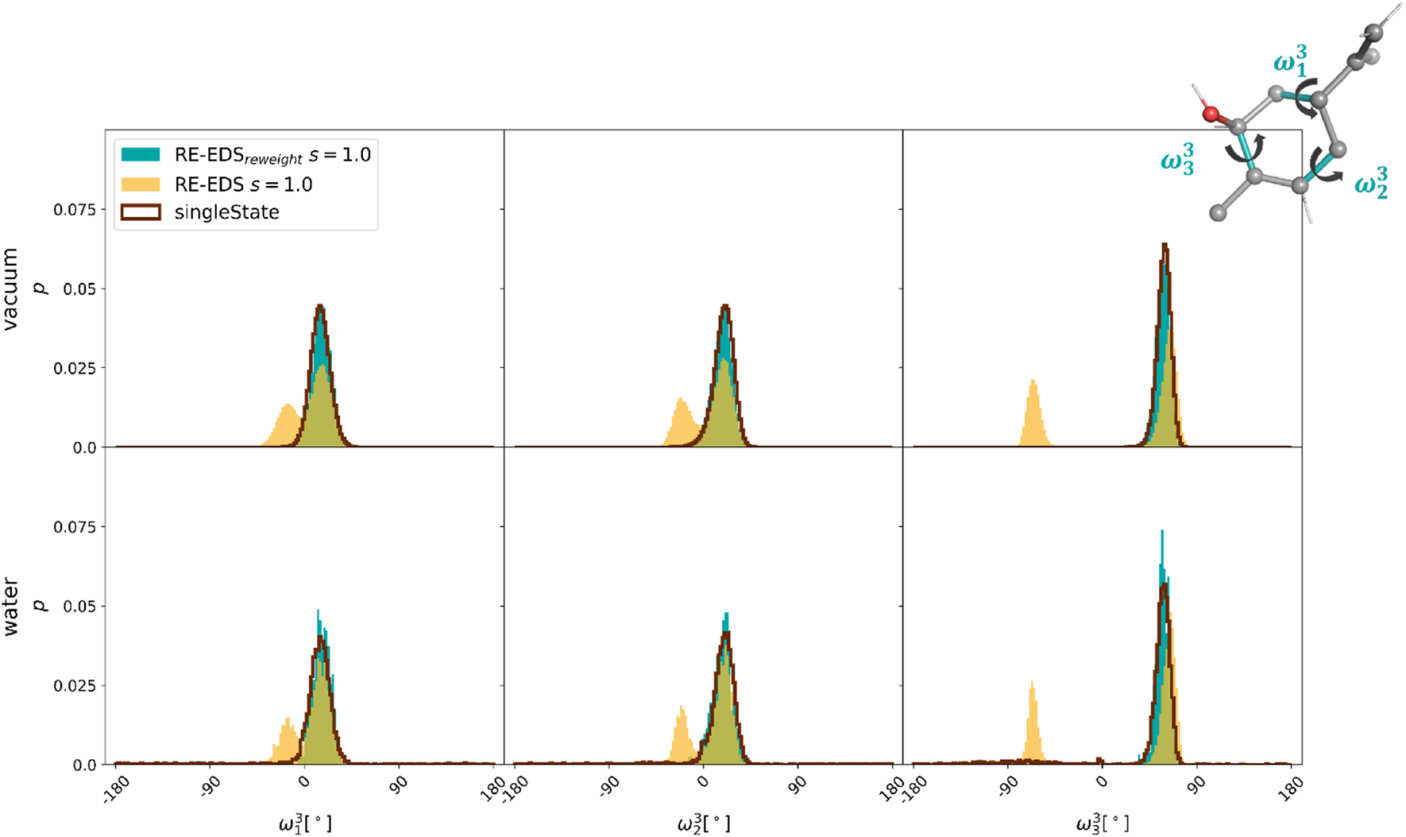
Comparison of the normalized torsional angle distributions of the three pseudo torsional angles of the cyclohexane ring of molecule **3** in the RE-EDS simulation at $s=1.0$ (filled) and in plain MD simulations (dark red line) in vacuum (top) and in water (bottom). Both the raw (yellow) and the reweighted (with $e^{\beta (V_R - V_i)}$, cyan) distributions are shown for RE-EDS.

The analyses of the conformational sampling effect of the applied distance restraints in the RE-EDS simulations of subset B are shown in Figs. S14 and S15 in the Supporting Information. Again the effect is negligible. The reweighted distributions of the three pseudo torsional angles of the cyclohexane ring of molecule **3** agree very well with the distributions from plain MD simulations (Fig. 15). The second peak visible in the raw RE-EDS simulations (i.e. not reweighted) comes from the frames where molecule **3** is in the dummy state (Fig. S16 in the Supporting Information).

## Conclusions

In this work, we presented an efficient greedy algorithm for the (locally) optimal placement of distance restraints in free-energy calculations performed with the linked dual topology approach. Linked dual topologies have the advantage that larger transformations can be simulated in a straightforward manner (e.g. no soft bonds are required), while reducing the sampling complexity. With the developed RestraintMaker Python package, distance restraint sets can be selected from a script or at GUI level, and written out in the GROMOS and GROMACS formats or in JSON format. The greedy algorithm is a graph-based approach and can be straightforwardly applied to molecules with (semi)rigid cores (typically aromatic or aliphatic rings). The cores do not necessarily have to be the same for all molecules, allowing for more complex transformations between ligands. The only required user inputs are the number of restraints $n_\text {res}$ to be selected and the maximum distance between the restrained atoms ($d_\text {res}$) . The performance of the algorithm was evaluated using toy systems (particle clouds) and compared to two brute-force approaches. In view of the results, the greedy algorithm represents a good trade-off between computing time and accuracy.

RestraintMaker was used to select optimal distance restraints for the calculation of relative hydration free energies with both TI (pairwise) and RE-EDS (multi-state). In all cases, good agreement between the different free-energy methods and with experiment was observed. Detailed analysis of the conformational sampling also indicated that the effect of the possible distortions induced by the distance restraints on the conformations is negligible. Even when restraining the benzene core and the cyclohexane core of two molecules together, accurate free-energy differences were obtained and the distributions of the pseudo torsional angles of the cyclohexane ring were nearly identical with those from plain MD simulations. The results obtained with RE-EDS highlight the superior sampling efficiency of the method. The application of RestraintMaker to estimate binding free energies with RE-EDS is currently ongoing and will be presented in future work.

## Supplementary Information

Below is the link to the electronic supplementary material.

Supplementary file1 (PDF 2849 kb)

## Data Availability

The RestraintMaker code can be downloaded from https://github.com/rinikerlab/restraintmaker. The Python code for the RE-EDS workflow is provided on Github https://github.com/rinikerlab/reeds and can be used with the current version of GROMOS, freely available from http://www.gromos.net. The input files for the simulations can be retrieved from https://github.com/rinikerlab/restraintmaker/tree/main/examples/publication.
