# Supplementary material for: RestraintMaker: a graph-based approach to select distance restraints in free-energy calculations with dual topology
Source: J Comput Aided Mol Des. 2022 Mar 22;36(3):175–92. doi: 10.1007/s10822-022-00445-6 (PMC8994745; doi:10.1007/s10822-022-00445-6)
Supplement: Supplementary file 1 — Supplementary file1 (PDF 2849 kb) [file 10822_2022_445_MOESM1_ESM.pdf]

SUPPORTING INFORMATION
RestraintMaker: A Graph-Based Approach to Select Distance Restraints in Free-Energy Calculations with Dual Topology

Benjamin Ries,[a] Salomé Rieder,[a] Clemens Rhiner,[a] Philippe H. Hünenberger,*[a] Sereina Riniker*[a]

[a] *Laboratory of Physical Chemistry, ETH Zürich, Vladimir-Prelog-Weg 2, 8093 Zürich, Switzerland. E-mail: sriniker@ethz.ch*

Contents

1 Ligands **S2**

2 Selected Distance Restraints **S2**

3 Calculation of Relative Hydration Free Energies **S5**
3.1 TI Calculations for Full Set of 16 Molecules S5
3.2 RE-EDS Simulations S10
3.2.1 Subset A S10
3.2.2 Subset B S14

1 Ligands

Table S1: Identifier of the ATB server [1], IUPAC name, and canonical SMILES for the 16 molecules with experimental hydration free energies.

Ligand	Identifier	IUPAC name	Canonical SMILES (RDKit [2])
1	_O6T	1,2-dimethoxybenzene	COc1ccccc1OC
2	_O70	(2R,5R)-2-methyl-5-prop-1-en-2-ylcyclohexan-1-one	C=C(C)[C@@H]1CC[C@@H](C)C(=O)C1
3	_O71	(1S,5R)-2-methyl-5-prop-1-en-2-ylcyclohex-2-en-1-ol	C=C(C)[C@@H]1CC=C(C)[C@@H](O)C1
4	_P8I	cyclopentanone	O=C1CCCC1
5	6J29	1-amino-4-hydroxyanthracene-9,10-dione	Nc1ccc(O)c2c1C(=O)c1ccccc1C2=O
6	6KET	3-methoxyphenol	COc1cccc(O)c1
7	8018	(1R,2S,3R,4R,6S,7S)-1,3,4,7,8,9,10,10-octachlorotricyclo[5.2.1.02,6]dec-8-ene	ClC1=C(Cl)[C@]2(Cl)[C@H]3C[C@@H](Cl)[C@H](Cl)[C@H]3[C@@]1(Cl)C2(Cl)Cl
8	E1VB	[1,2,2-Trifluoroethoxy]benzene	FC(F)[C@@H](F)Oc1ccccc1
9	F313	4-methoxyaniline	COc1ccc(N)cc1
10	G078	1,4-dimethylnaphthalene	Cc1ccc(C)c2ccccc12
11	G277	cyclohexa-2,5-diene-1,4-dione	O=C1C=CC(=O)C=C1
12	M030	1,3,5-trimethylbenzene	Cc1cc(C)cc(C)c1
13	M097	2-chloroaniline	Nc1ccccc1Cl
14	M218	N-methylaniline	CNc1ccccc1
15	S002	bromomethylbenzene	BrCc1ccccc1
16	TVVS	pyridine-4-carbaldehyde	O=Cc1ccncc1

2 Selected Distance Restraints

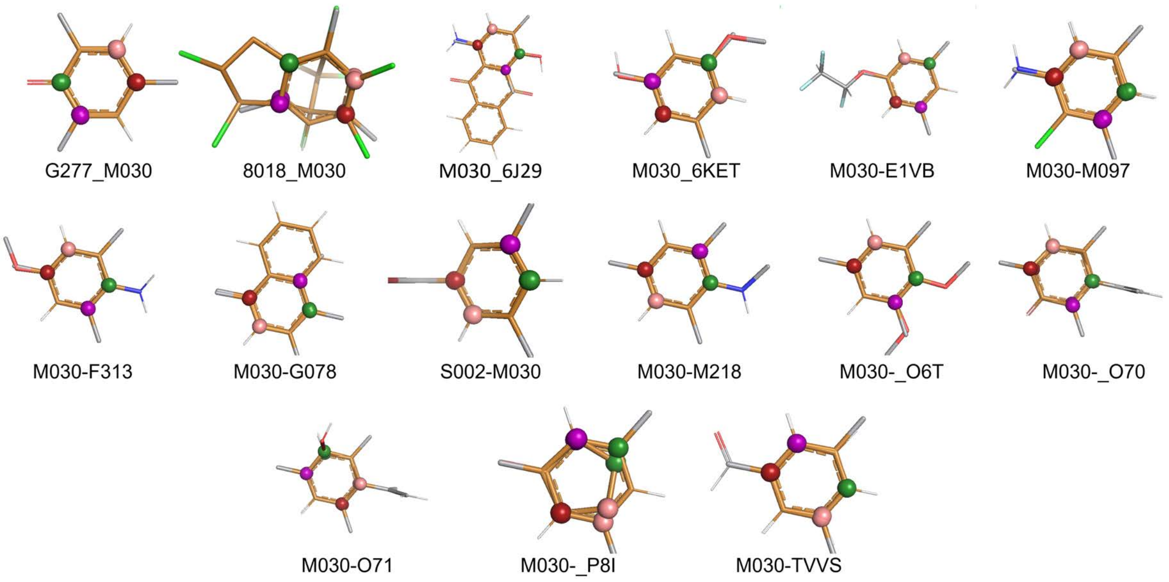

Figure S1: Selected distance restraints (colored spheres) for the 15 pairwise TI calculations with molecule 12 (i.e. 1,3,5-timethylbenzene) as the central molecule (see Main Article Figure 6). For each pair, four distance restraints were determined with the greedy algorithm.

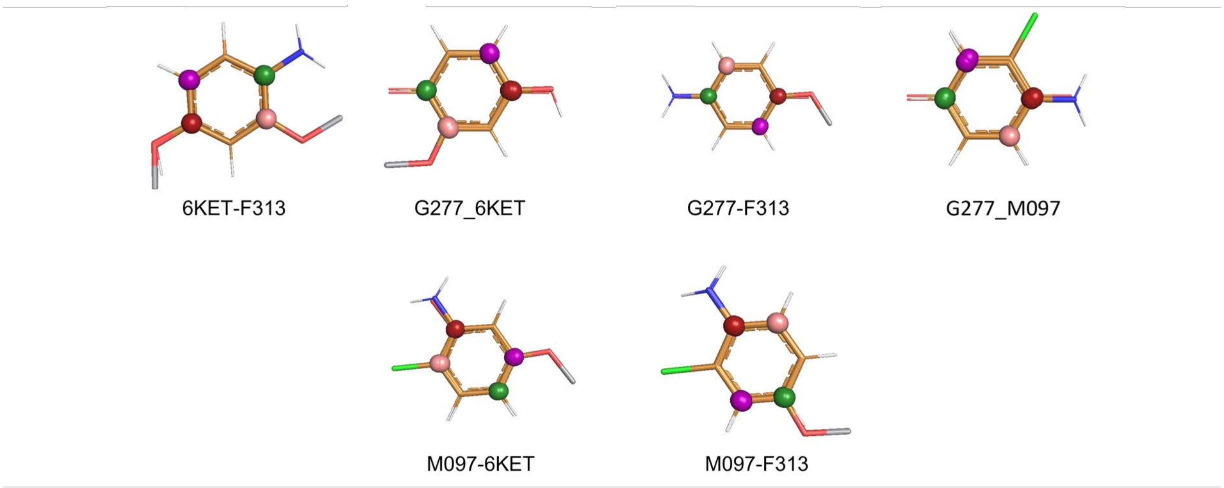

Figure S2: Selected distance restraints (colored spheres) for the TI simulations of subset A (see Main Article Figure 7a). For each pair, four distance restraints were determined with the greedy algorithm.

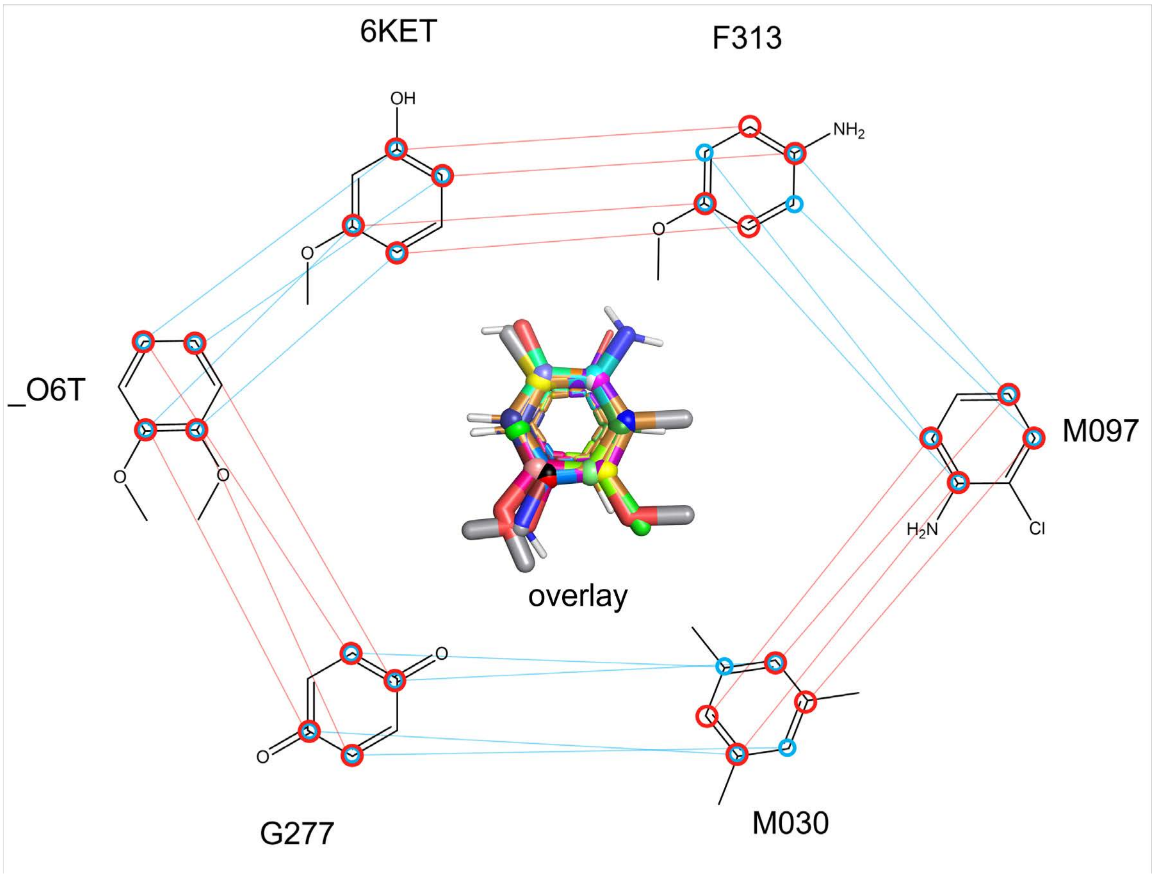

Figure S3: Selected distance restraints (colored spheres) for the RE-EDS simulations of subset A (see Main Article Figure 7a). For each pair, four distance restraints were determined with the greedy algorithm. The lines indicate which ligands were connected with each other.

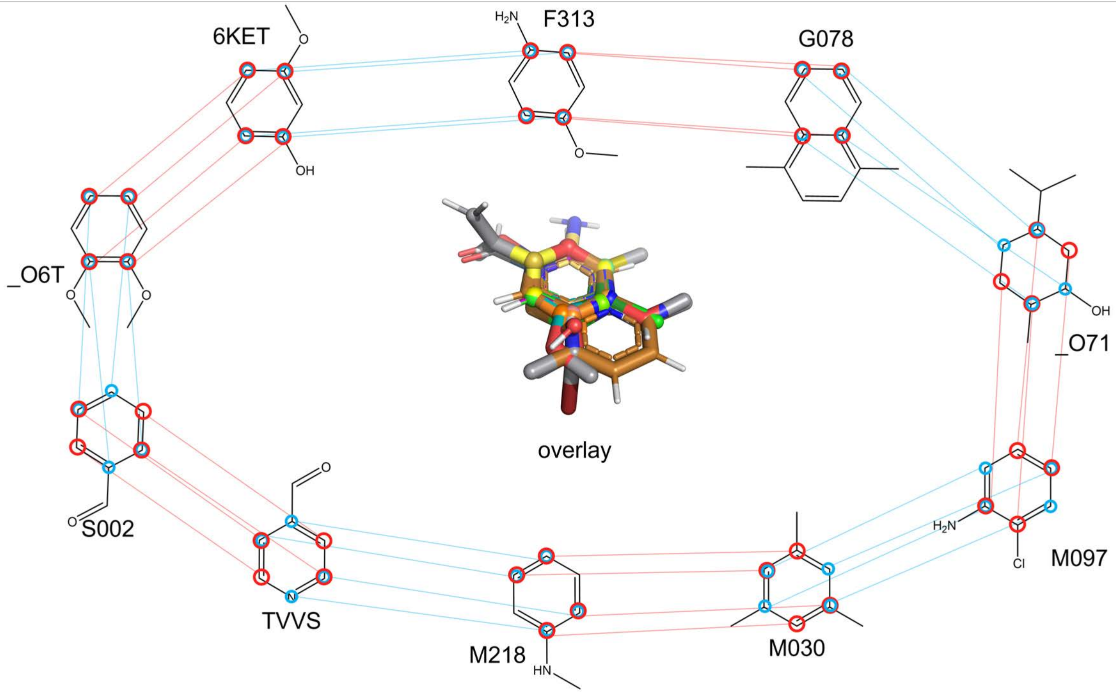

Figure S4: Selected distance restraints (colored spheres) for the RE-EDS simulations of subset B (see Main Article Figure 7b. For each pair, four distance restraints were determined with the greedy algorithm. The lines indicate with ligands were connected with each other.

3 Calculation of Relative Hydration Free Energies

3.1 TI Calculations for Full Set of 16 Molecules

Table S2: $\Delta\Delta G_{\text{hyd}}$ for the 16 small molecules from experiment, the absolute free-energy calculations with TI taken from the ATB server [1] (TI, abs), and the pairwise relative free-energy calculations with TI and linked dual topology (TI, rel). The uncertainty estimate was calculated via Gaussian error propagation of the provided errors. The experimental uncertainty for molecule 11 was set to a default value of 2.5 kJ/mol [3], as the uncertainty was not reported in the original source [4]. The RMSE and its uncertainty were estimated with a 100 fold bootstrap approach. The accumulated simulation time is split into preparation (pre-processing, equilibration) and production run. The data is displayed graphically in Main Article Figure 9

Ligands i	j	Experiment [kJ/mol]	$\Delta\Delta G_{\text{hyd}}^{\text{TI,direct}}$ [kJ/mol]	$\Delta\Delta G_{\text{hyd}}^{\text{TI,indirect}}$ [kJ/mol]
1	12	18.5 ± 2.5 [5, 6]	26.2 ± 0.8	17.5 ± 0.7
2	12	11.9 ± 2.7 [5, 6]	14.4 ± 0.7	8.6 ± 0.6
3	12	14.8 ± 3.1 [5, 6]	22.4 ± 0.7	15.2 ± 0.9
4	12	15.9 ± 3.5 [6]	15.3 ± 0.6	9.4 ± 0.4
5	12	36.1 ± 2.8 [5, 6]	48.2 ± 0.4	46.9 ± 0.8
6	12	28.1 ± 3.5 [6]	36.1 ± 0.6	30.5 ± 0.5
7	12	10.6 ± 2.5 [6, 7]	22.8 ± 0.5	9.5 ± 0.9
8	12	1.6 ± 3.5 [3, 6]	5.2 ± 0.7	3.0 ± 1.2
9	12	27.5 ± 3.5 [6]	30.2 ± 0.6	25.7 ± 0.5
10	12	8.0 ± 3.5 [6]	6.5 ± 0.6	5.9 ± 0.5
11	12	20.4 ± 3.5 [4, 6]	17.0 ± 0.6	14.8 ± 0.39
12	13	16.8 ± 3.5 [6]	18.6 ± 0.6	16.6 ± 0.5
12	14	15.9 ± 3.5 [6]	24.9 ± 0.6	20.5 ± 0.5
12	15	6.2 ± 2.6 [6, 8]	14.9 ± 0.7	10.3 ± 0.4
12	16	25.5 ± 3.5 [6]	26.1 ± 0.6	24.0 ± 0.4
RMSE			6.7 ± 0.3	4.1 ± 0.3
MAE			5.5 ± 3.9	3.1 ± 2.7
r_{Spearman}			0.84	0.87
$t_{preparation}$				630 ns
$t_{production}$			$112 - 272\ ns$	3150 ns

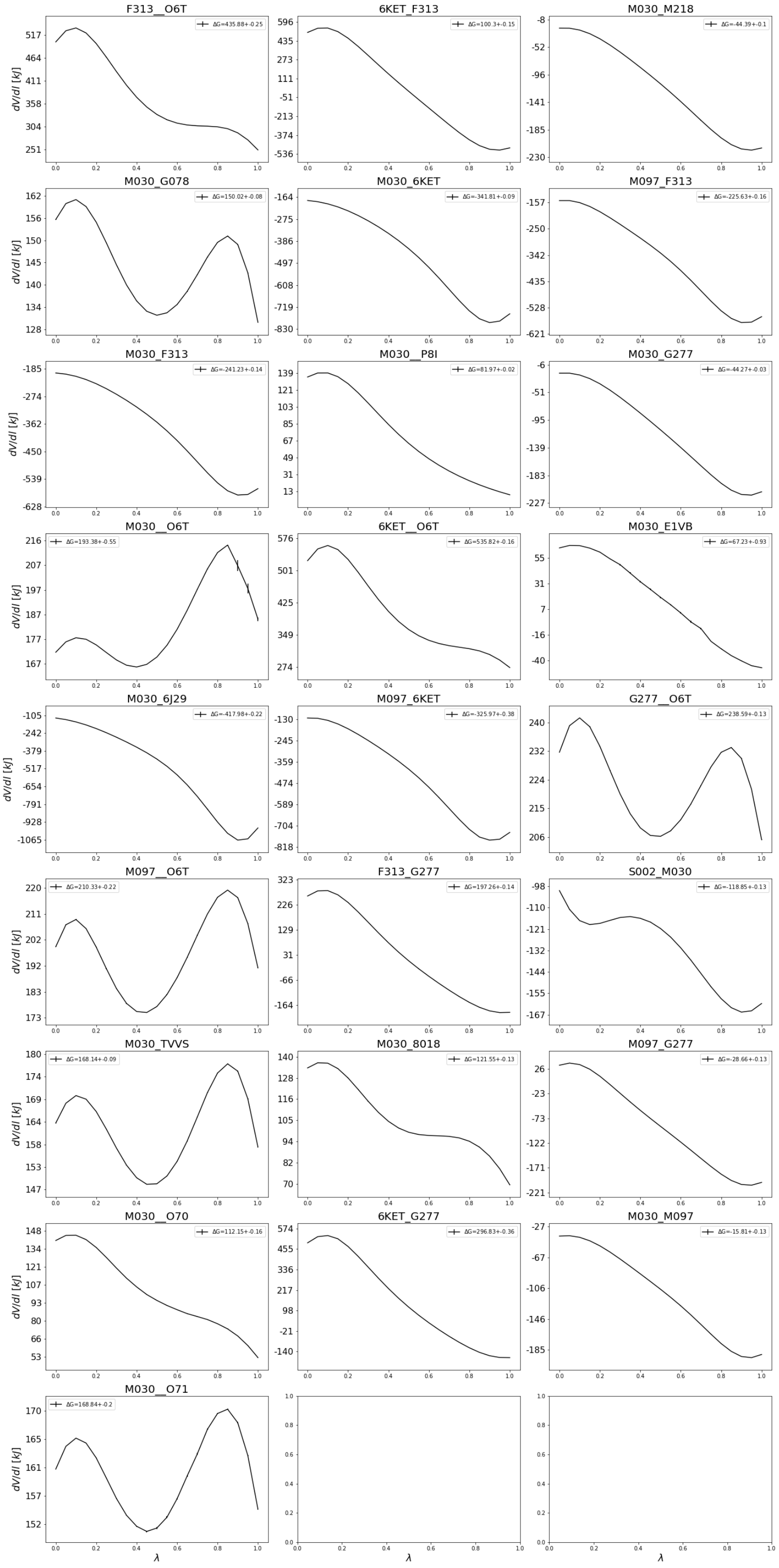

Figure S5: $\left\langle \frac{\partial V(\lambda)}{\partial \lambda} \right\rangle_\lambda$ as a function of λ for the 15 pairwise TI calculations in vacuum. The production run was 5 ns per λ-point.

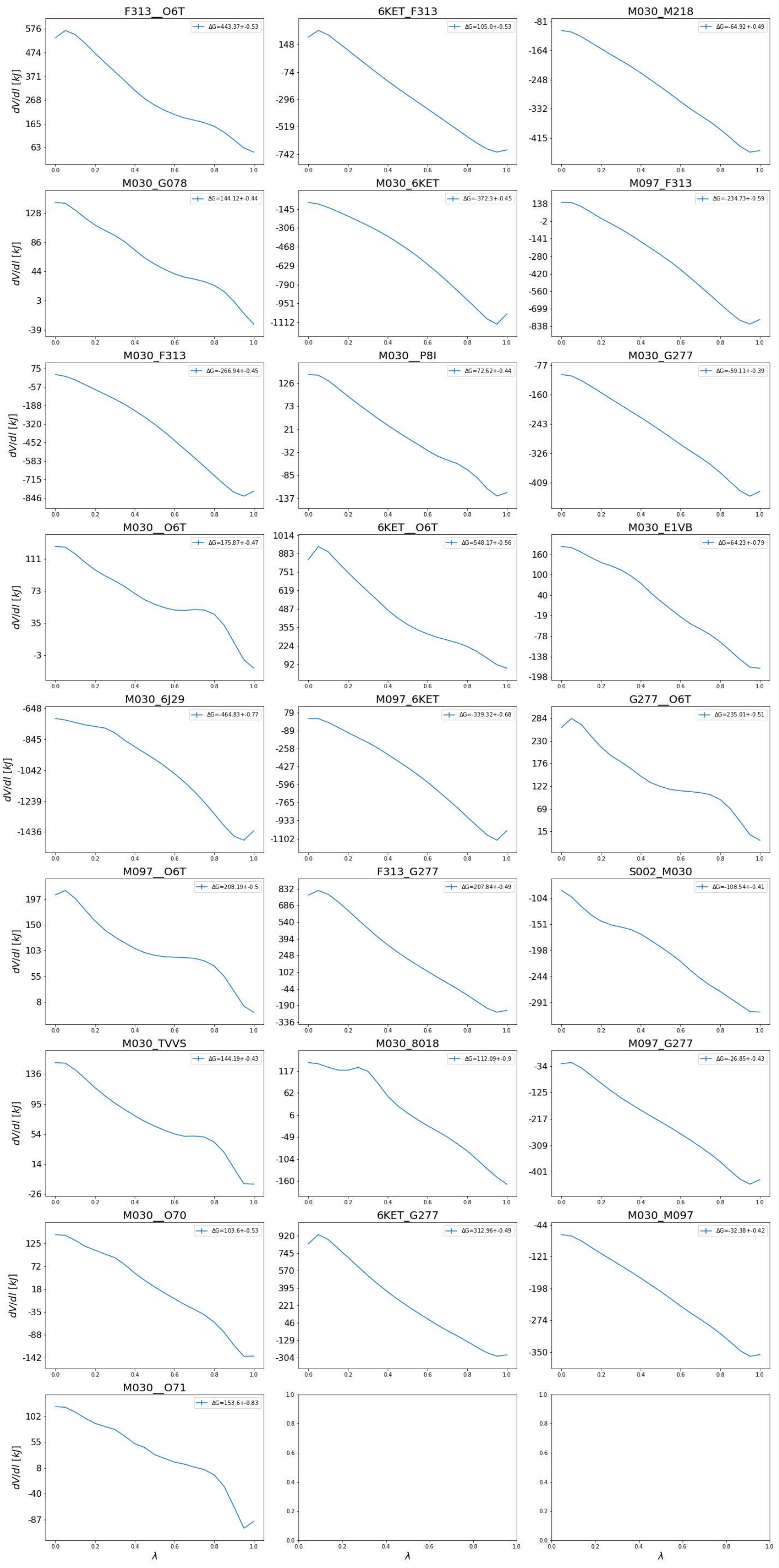

Figure S6: $\left\langle \frac{\partial V(\lambda)}{\partial \lambda} \right\rangle_\lambda$ as a function of λ for the 15 pairwise TI calculations in water. The production run was 5 ns per λ-point.

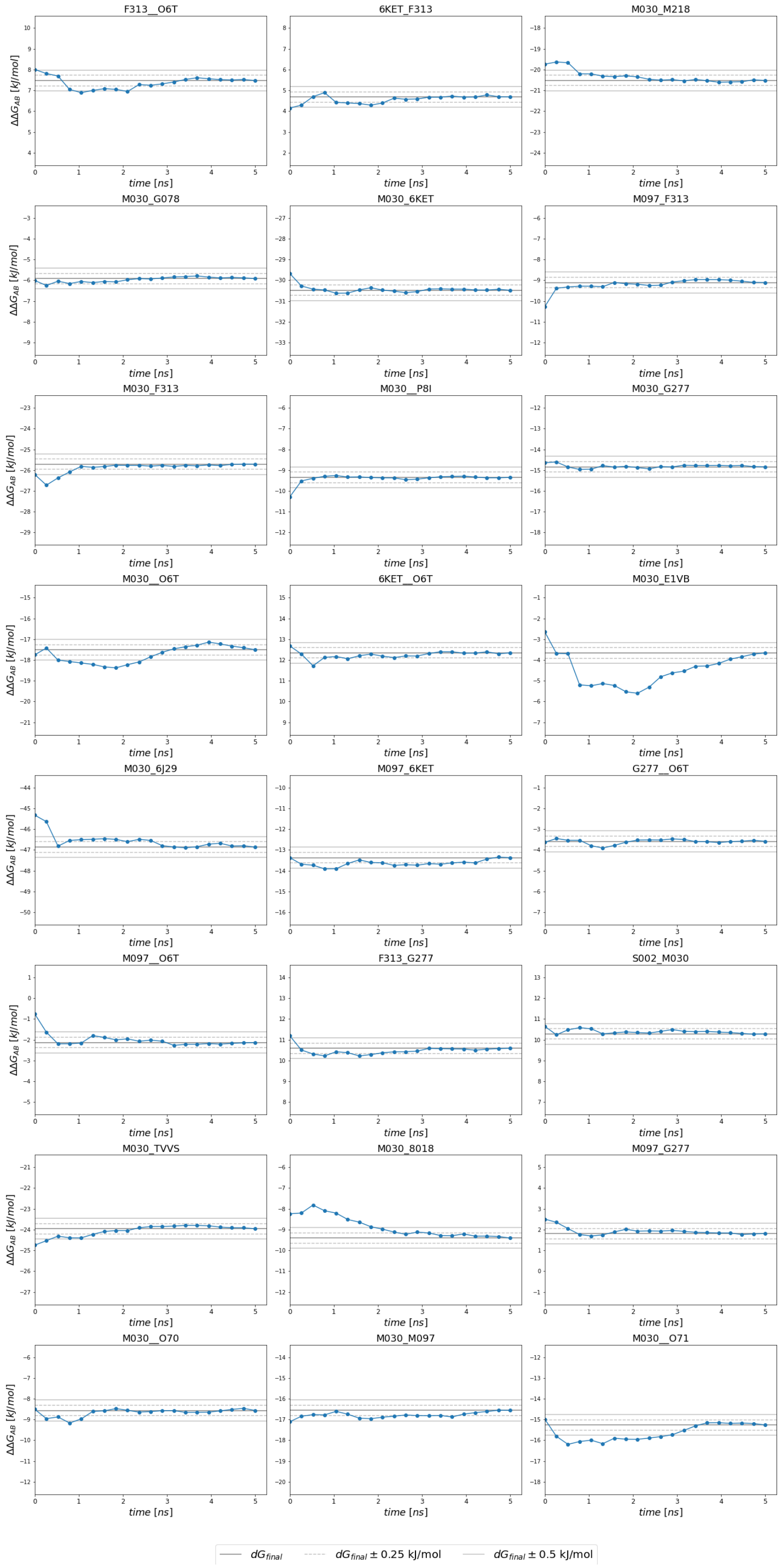

Figure S7: Convergence of $\Delta\Delta G_{\mathrm{hyd}}$ as a function of the simulation time per λ-point for the 15 pairwise TI calculations.

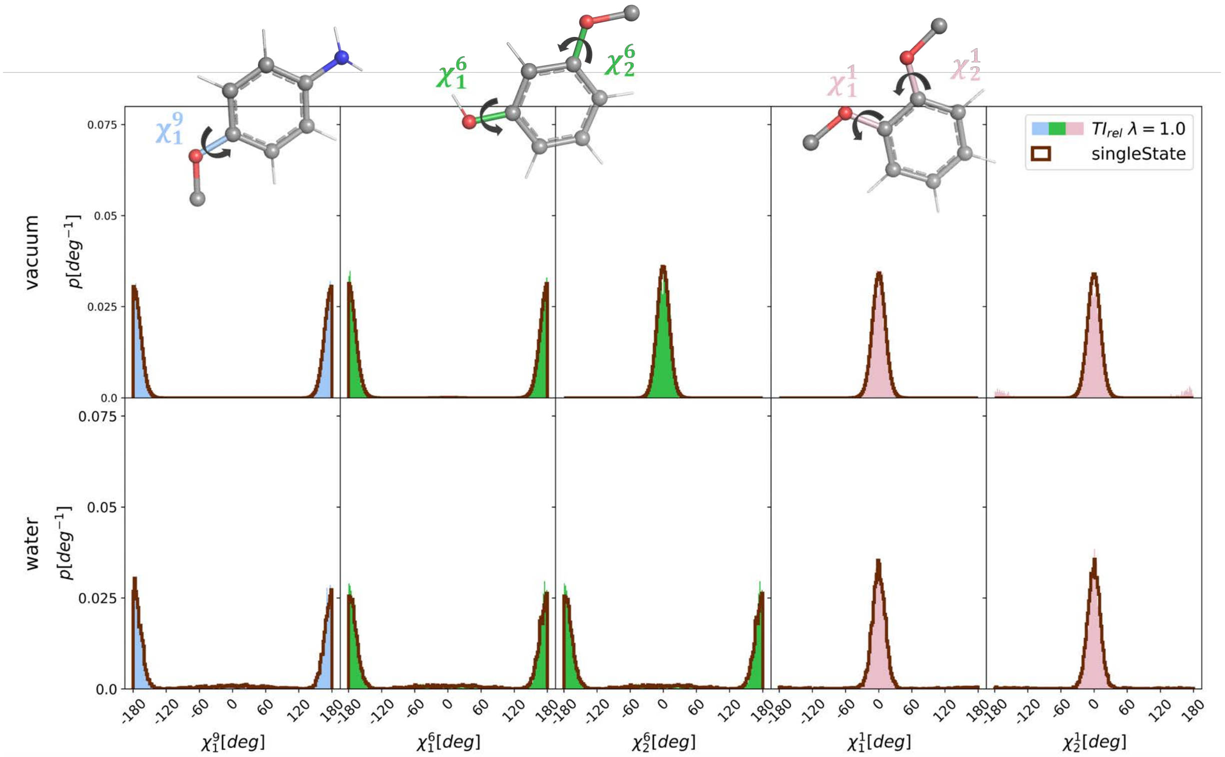

Figure S8: Comparison of the normalized torsional angle distributions of the substituents of molecules 9 (blue), 6 (green), and 1 (pink) in the simulation at $\lambda = 1.0$ (filled) and in plain MD simulations (dark red line) in vacuum (top) and in water (bottom).

3.2 RE-EDS Simulations

3.2.1 Subset A

Table S3: $\Delta\Delta G_{\text{hyd}}$ for the six molecules in subset A from experiment, the absolute free-energy calculations with TI taken from the ATB server [1] (TI, direct), the pairwise relative calculations with TI and linked dual topology (TI, indirect), and the multi-state relative free-energy calculations with RE-EDS and linked dual topology (RE-EDS, indirect). For the relative calculations with TI, the results for both the full graph and the minimal five molecule pairs are given. The uncertainty estimate was calculated via Gaussian error propagation of the provided errors. The experimental uncertainty for molecule 11 was set to a default value of 2.51 kJ/mol [3], as the uncertainty was not reported in the original source [4]. The RMSE and its uncertainty were estimated with a 100 fold bootstrap approach. The accumulated simulation time is split into preparation (pre-processing, equilibration) and production run. The data is displayed graphically in Main Article Figure 13.

Ligands i	j	Experiment [kJ/mol]	$\Delta\Delta G_{\text{hyd}}^{\text{TI,direct}}$ [kJ/mol]	$\Delta\Delta G_{\text{hyd}}^{\text{TI,indirect}}$ (minimal) [kJ/mol]	$\Delta\Delta G_{\text{hyd}}^{\text{TI,indirect}}$ (full) [kJ/mol]	$\Delta\Delta G_{\text{hyd}}^{\text{RE-EDS,indirect}}$ [kJ/mol]
1	6	-9.8 ± 2.5 [5, 6]	-9.9 ± 0.9	-13.0 ± 0.6	-12.4 ± 0.6	-11.6 ± 0.4
1	9	-9.0 ± 2.5 [5, 6]	-4.0 ± 0.7	-8.2 ± 0.6	-7.5 ± 0.6	-6.9 ± 0.4
1	11	-1.9 ± 2.5[4, 5]	9.2 ± 0.9	2.7 ± 0.5	3.6 ± 0.5	3.8 ± 0.3
1	12	18.5 ± 2.5 [5, 6]	26.2 ± 0.8	17.5 ± 0.7	17.5 ± 0.7	18.8 ± 0.3
1	13	1.8 ± 2.5 [5, 6]	7.6 ± 0.9	0.9 ± 0.5	2.1 ± 0.5	2.2 ± 0.3
6	9	0.8 ± 3.5 [6]	5.9 ± 0.7	4.8 ± 0.6	4.7 ± 0.9	4.8 ± 0.3
6	11	7.9 ± 3.5 [4, 6]	19.1 ± 0.7	15.6 ± 0.6	16.1 ± 1.1	15.4 ± 0.2
6	12	28.3 ± 3.5 [6]	36.1 ± 0.6	30.5 ± 0.5	30.5 ± 0.5	30.4 ± 0.3
6	13	11.5 ± 3.5 [6]	17.5 ± 0.7	13.9 ± 0.8	13.4 ± 0.8	13.8 ± 0.3
9	11	7.1 ± 3.5 [4, 6]	13.2 ± 0.6	10.9 ± 0.5	10.6 ± 0.5	10.6 ± 0.2
9	12	27.5 ± 3.5 [6]	30.2 ± 0.6	25.7 ± 0.5	25.7 ± 0.5	25.7 ± 0.3
9	13	10.8 ± 3.5 [6]	11.6 ± 0.7	9.1 ± 0.6	9.1 ± 0.6	9.0 ± 0.2
11	12	20.4 ± 3.5 [4, 6]	17.0 ± 0.6	14.8 ± 0.4	14.8 ± 0.4	15.1 ± 0.2
11	13	3.7 ± 3.5 [4, 6]	-1.6 ± 0.7	-1.7 ± 0.4	-1.8 ± 0.5	-1.6 ± 0.2
12	13	-16.8 ± 3.5 [6]	-18.6 ± 0.6	-16.6 ± 0.4	-16.6 ± 0.5	-16.6 ± 0.2
RMSE			6.2 ± 0.3	3.7 ± 0.1	3.8 ± 0.2	3.6 ± 0.3
MAE			5.3 ± 3.2	3.0 ± 2.1	3.0 ± 2.2	2.9 ± 2.1
r_{Spearman}			0.92	0.93	0.93	0.93
$t_{preparation}$				215 ns	660 ns	222 ns
$t_{production}$			$42 - 102\ ns$	1050 ns	3150 ns	36 ns

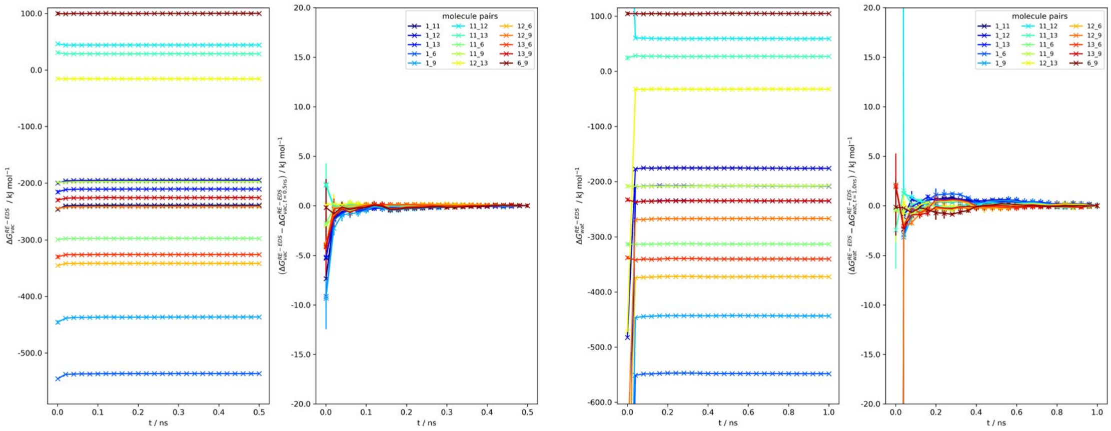

Figure S9: Convergence of ΔG_{vac} and ΔG_{wat} as a function of the simulation time for the RE-EDS simulation of subset A ($s = 1.0$) in vacuum (left) and in water (right).

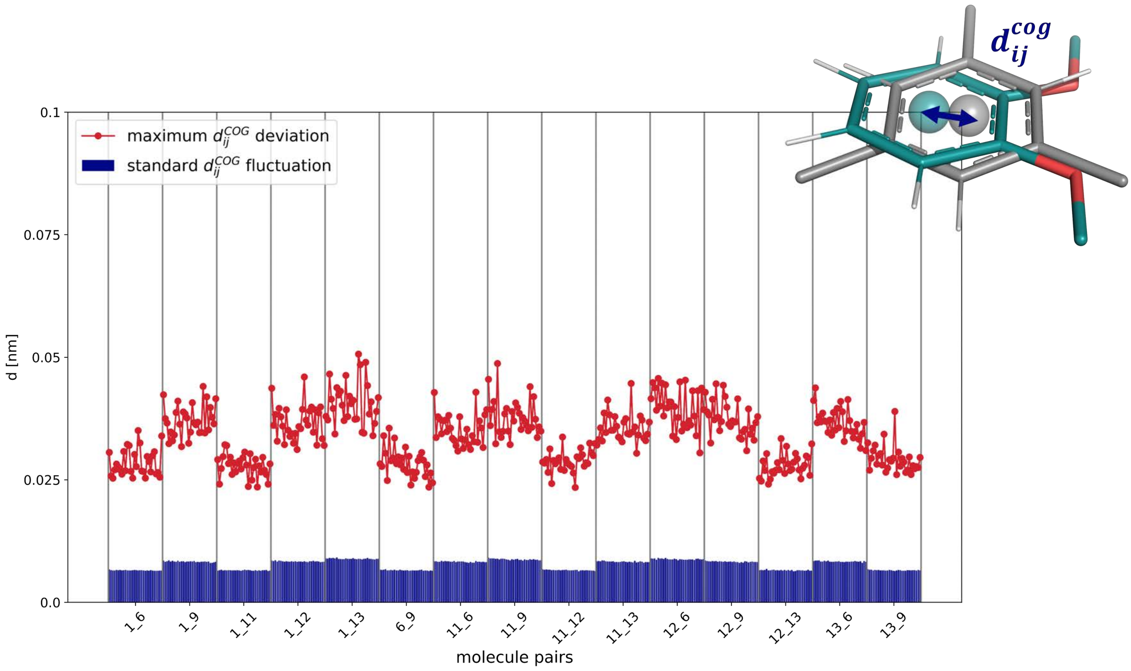

Figure S10: Standard deviation of the distance distribution (blue) and maximum distance (red) between the COGs of the central rings of the molecule pairs in the RE-EDS simulations of subset A in water ($s = 1.0$). The COG was calculated for the restrained atoms in the rings. The horizontal axis shows for each molecule pair the different s-values between 1.0 and 0.001.

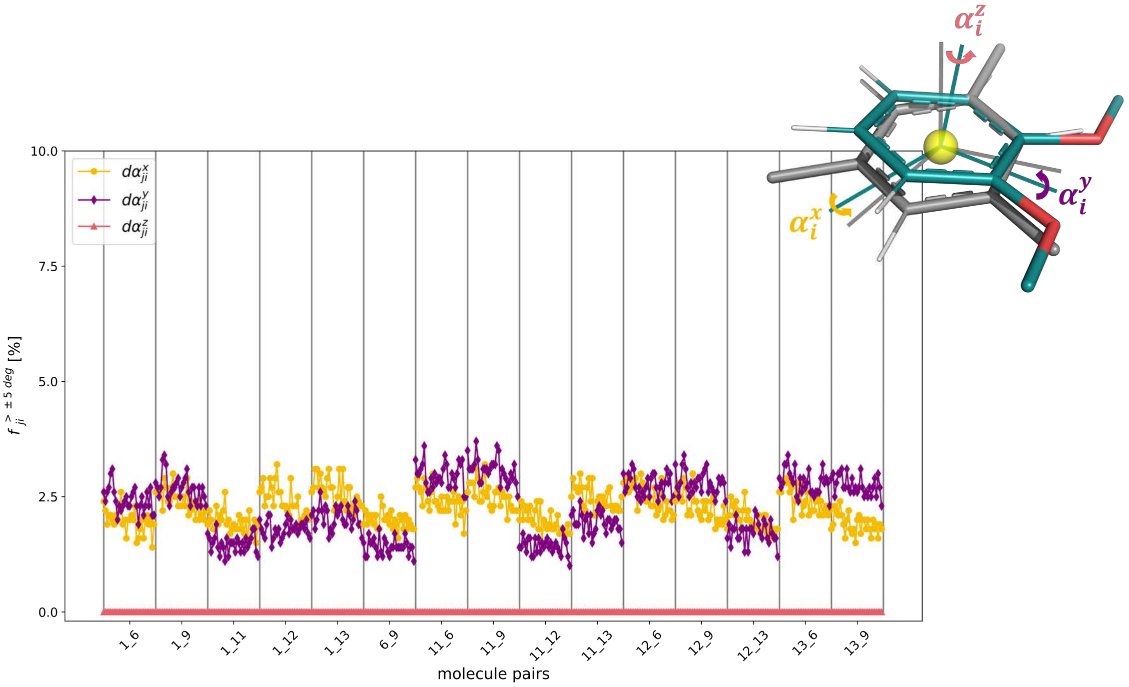

Figure S11: Fraction of frames in the RE-EDS simulations of subset A in water ($s = 1.0$), in which the relative rotation around the x-axis (yellow), y-axis (purple), and z-axis (red) of the central rings of the molecule pair exceeds $5°$. The horizontal axis shows for each molecule pair the different s-values between 1.0 and 0.001.

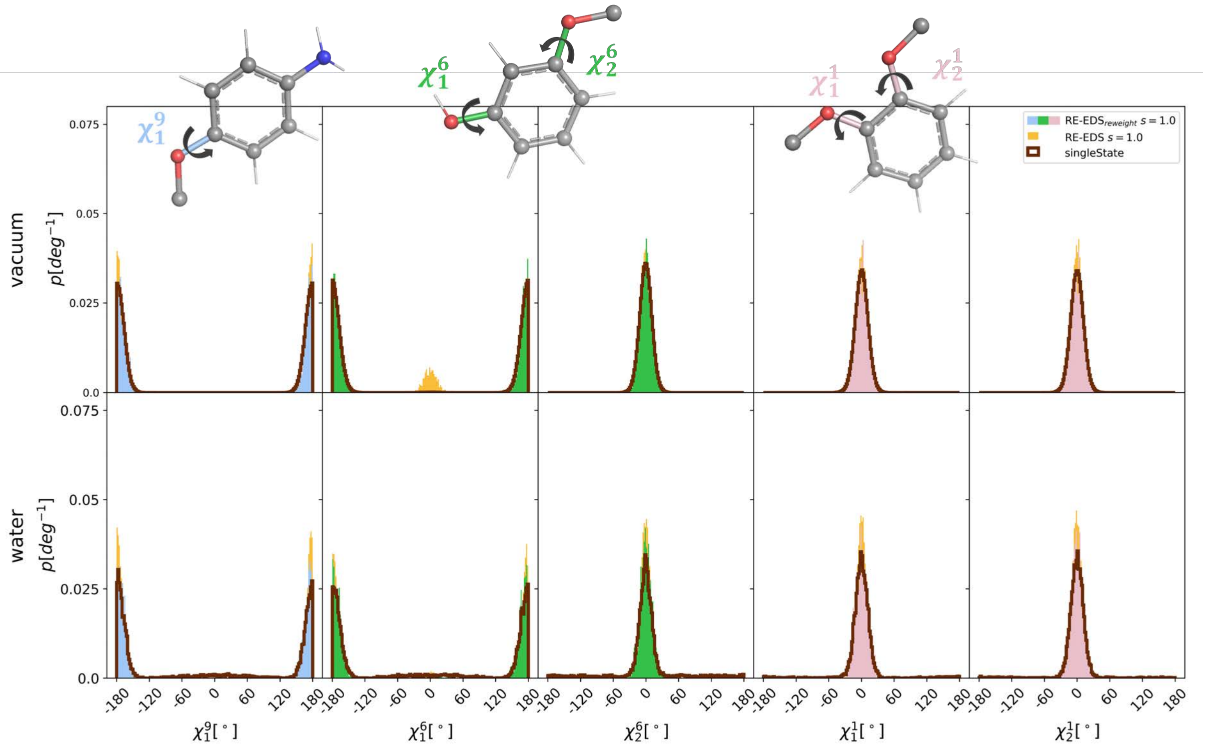

Figure S12: Comparison of the normalized torsional angle distributions of the substituents of molecules 9 (blue), 6 (green), and 1 (pink) in the RE-EDS simulation of subset A at $s = 1.0$ (filled) and in plain MD simulations (dark red line) in vacuum (top) and in water (bottom). Each frame was reweighted with $e^{+\beta(V_R - V_i)}$.

3.2.2 Subset B

Table S4: $\Delta\Delta G_{\text{hyd}}$ for the six molecules in subset A from experiment, the absolute free-energy calculations with TI taken from the ATB server [1] (TI, direct), the pairwise relative calculations with TI and linked dual topology (TI, indirect), and the multi-state relative free-energy calculations with RE-EDS and linked dual topology (RE-EDS, indirect). For the relative calculations with TI, the results from part 1 (16 molecules) were used. The uncertainty estimates were obtained as in Table S3. The accumulated simulation time is split into preparation (pre-processing, equilibration) and production run. The data is displayed graphically in Main Article Figure 14.

Ligands i	j	Experiment [kJ/mol]	$\Delta\Delta G_{\text{hyd}}^{\text{TI,direct}}$ [kJ/mol]	$\Delta\Delta G_{\text{hyd}}^{TI,indirect}$ (minimal) [kJ/mol]	$\Delta\Delta G_{\text{hyd}}^{\text{RE-EDS,indirect}}$ [kJ/mol]
1	3	3.7 ± 1.8 [5]	3.8 ± 1.0	2.3 ± 0.5	3.2 ± 0.5
1	15	12.3 ± 0.9 [5, 8]	11.3 ± 0.8	7.2 ± 1.1	8.8 ± 0.2
1	12	18.5 ± 2.5 [5, 6]	26.2 ± 0.8	17.5 ± 0.7	18.9 ± 0.1
1	6	−9.6 ± 2.5 [5, 6]	−9.8 ± 0.9	−12.4 ± 0.6	−11.5 ± 0.1
1	9	−9.0 ± 2.5 [5, 6]	−3.4 ± 0.9	−7.5 ± 0.6	−7.4 ± 0.2
1	13	1.8 ± 2.5 [5, 6]	7.6 ± 0.9	2.1 ± 0.5	1.7 ± 0.2
1	10	10.5 ± 2.5 [5, 6]	19.8 ± 0.9	11.6 ± 0.5	13.1 ± 0.1
1	14	2.7 ± 2.5 [5, 6]	1.3 ± 0.9	−3.0 ± 1.2	−1.7 ± 0.1
1	16	−7.0 ± 2.5 [5]	0.1 ± 0.9	−6.4 ± 1.1	−4.8 ± 0.4
3	15	8.6 ± 2.0 [5, 8]	7.4 ± 0.9	4.9 ± 1.3	5.7 ± 0.5
3	12	14.8 ± 3.1 [5, 6]	22.4 ± 0.7	15.2 ± 0.9	15.8 ± 0.5
3	6	−13.5 ± 3.1 [5, 6]	−13.7 ± 0.8	−15.3 ± 0.4	−14.6 ± 0.5
3	9	−12.7 ± 3.1 [5, 6]	−7.8 ± 0.8	−10.5 ± 0.4	−10.4 ± 0.5
3	13	−1.9 ± 3.1 [5, 6]	3.8 ± 0.8	−1.3 ± 1.4	−1.5 ± 0.5
3	10	6.8 ± 3.1 [5, 6]	15.9 ± 0.8	9.3 ± 0.4	9.9 ± 0.5
3	14	−1.0 ± 3.1 [5, 6]	−2.6 ± 0.8	−5.3 ± 1.4	−4.8 ± 0.5
3	16	−10.7 ± 3.1 [5, 6]	−3.8 ± 0.8	−8.7 ± 1.3	−7.9 ± 0.6
15	12	6.2 ± 2.6 [6, 8]	14.9 ± 0.7	10.3 ± 0.4	10.1 ± 0.2
15	6	−22.1 ± 2.6 [6, 8]	−21.2 ± 0.8	−20.2 ± 0.9	−20.3 ± 0.2
15	9	−21.3 ± 2.6 [6, 8]	−15.2 ± 0.8	−15.4 ± 0.9	−16.1 ± 0.3
15	13	−10.6 ± 2.6 [6, 8]	−3.7 ± 0.8	−6.3 ± 0.1	−7.2 ± 0.3
15	10	−1.8 ± 2.6 [6, 8]	8.5 ± 0.8	4.4 ± 0.9	4.2 ± 0.2
15	14	−9.7 ± 2.6 [6, 8]	−10.0 ± 1.0	−10.2 ± 0.1	−10.5 ± 0.2
15	16	−19.3 ± 2.6 [6, 8]	−11.2 ± 0.8	−13.6 ± 0.0	−13.6 ± 0.5
12	6	−28.3 ± 3.5 [6]	−36.1 ± 0.6	−30.5 ± 0.5	−30.4 ± 0.1
12	9	−27.5 ± 3.5 [6]	−30.2 ± 0.6	−25.7 ± 0.5	−26.2 ± 0.2
12	13	−16.8 ± 3.5 [6]	−18.6 ± 0.6	−16.6 ± 0.5	−17.3 ± 0.2
12	10	−8.0 ± 3.5 [6]	−6.5 ± 0.6	−5.9 ± 0.5	−5.9 ± 0.1
12	14	−15.9 ± 3.5 [6]	−24.9 ± 0.6	−20.5 ± 0.5	−20.6 ± 0.1
12	16	−25.3 ± 3.5 [6]	−26.1 ± 0.6	−24.0 ± 0.4	−23.7 ± 0.4
6	9	0.8 ± 3.5 [6]	6.4 ± 0.7	4.7 ± 0.9	4.2 ± 0.2
6	13	11.5 ± 3.5 [6]	17.5 ± 0.8	13.4 ± 0.8	13.1 ± 0.2
6	10	20.3 ± 3.5 [6]	29.6 ± 0.8	24.6 ± 0.0	24.5 ± 0.1
6	14	12.4 ± 3.5 [6]	11.2 ± 0.8	10.0 ± 1.0	9.8 ± 0.1
6	16	2.8 ± 3.5 [6]	10.0 ± 0.8	6.5 ± 1.0	6.7 ± 0.4
9	13	10.8 ± 3.5 [6]	11.6 ± 0.7	9.1 ± 0.6	9.0 ± 0.3
9	10	19.5 ± 3.5 [6]	23.7 ± 0.6	19.8 ± 0.1	20.3 ± 0.2
9	14	11.7 ± 3.5 [6]	5.2 ± 0.6	5.2 ± 1.0	5.6 ± 0.2
9	16	2.8 ± 3.5 [6]	4.1 ± 0.6	1.8 ± 1.0	2.5 ± 0.4
13	10	8.7 ± 3.5 [6]	12.2 ± 0.7	10.7 ± 1.0	11.4 ± 0.2
13	14	0.9 ± 3.5 [6]	−6.3 ± 0.7	−4.0 ± 0.0	−3.3 ± 0.2
13	16	−8.8 ± 3.5 [6]	−7.5 ± 0.7	−7.4 ± 0.1	−6.5 ± 0.4
10	14	−7.8 ± 3.5 [6]	−18.5 ± 0.6	−14.6 ± 1.0	−14.7 ± 0.1
10	16	−17.5 ± 3.5 [6]	−19.7 ± 0.6	−18.1 ± 0.9	−17.8 ± 0.4
14	16	−9.7 ± 3.5 [6]	−1.2 ±	−3.4 ± 0.1	−3.1 ± 0.4
RMSE			5.8 ± 0.1	3.4 ± 0.1	3.3 ± 0.1
MAE			4.8 ± 3.3	2.8 ± 2.0	2.7 ± 1.8
r_{Spearman}			0.93	0.96	0.96
$t_{preparation}$				378 ns	418 ns
$t_{production}$			70 − 170 ns	1890 ns	212 ns

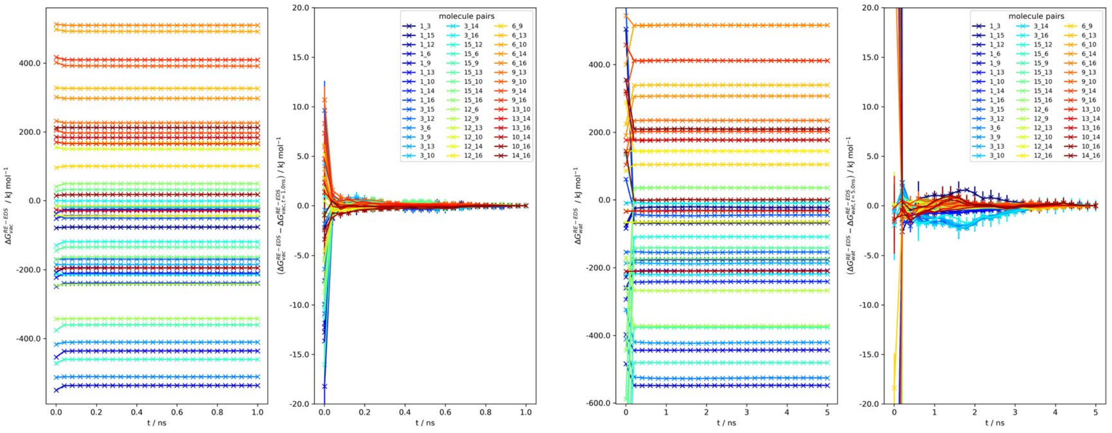

Figure S13: Convergence of ΔG_{vac} and ΔG_{wat} as a function of the simulation time for the RE-EDS simulation of subset B ($s = 1.0$) in vacuum (left) and in water (right).

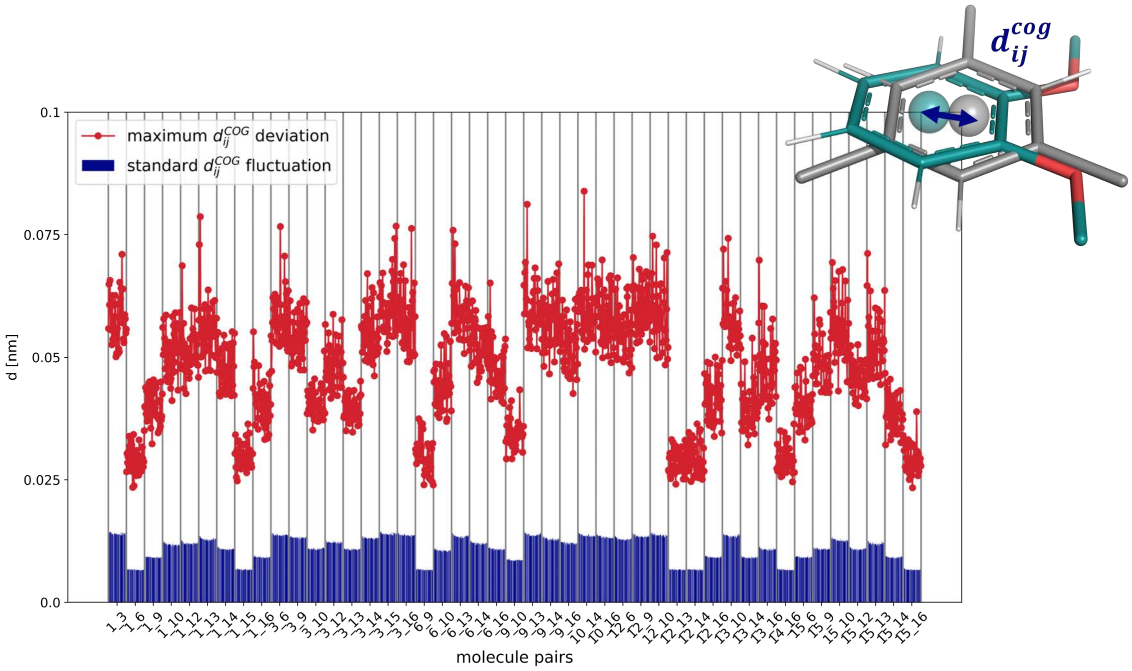

Figure S14: Standard deviation of the distance distribution (blue) and maximum distance (red) between the COGs of the central rings of the molecule pairs in the RE-EDS simulations of subset B in water ($s = 1.0$). The COG was calculated for the restrained atoms in the rings. The horizontal axis shows for each molecule pair the different s-values between 1.0 and 0.001.

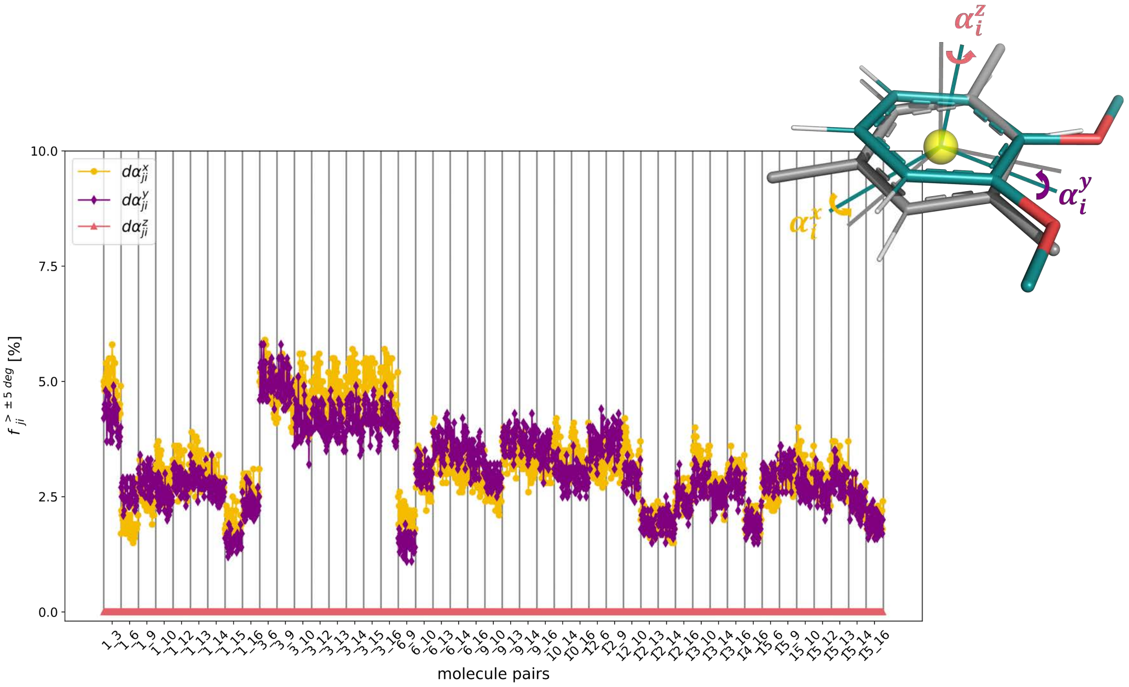

Figure S15: Fraction of frames in the RE-EDS simulations of subset B in water ($s = 1.0$), in which the relative rotation around the x-axis (yellow), y-axis (purple), and z-axis (red) of the central rings of the molecule pair exceeds 5°. The horizontal axis shows for each molecule pair the different s-values between 1.0 and 0.001.

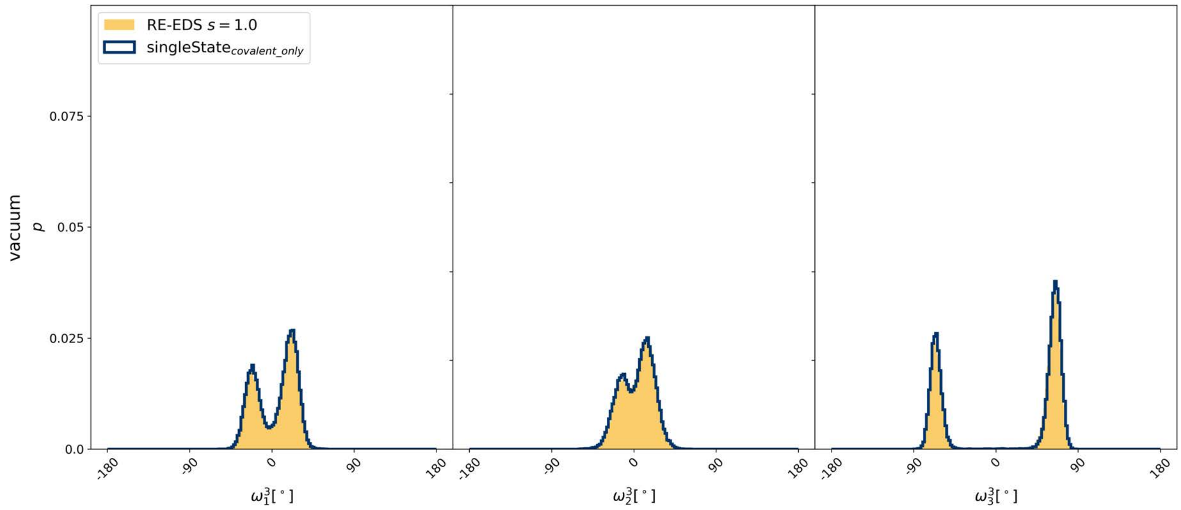

Figure S16: Comparison of the normalized torsional angle distributions of the three pseudo torsional angles of the cyclohexane ring of molecule **3** in a RE-EDS simulation with force constant $0\ \mathrm{kJ}/(\mathrm{mol{\cdot}nm}^2)$ at $s = 1.0$ (filled yellow) and in plain MD simulations in vacuum with only the covalent interactions turned on (dark blue lines). The distributions show that during the RE-EDS simulation, the same ring torsional configurations occur as when the non-bonded interactions are turned off in a single state simulation, even when the distance restraints are turned off.

References

[1] M. Stroet, B. Caron, K.M. Visscher, D.P. Geerke, A.K. Malde, A.E. Mark, J. Chem. Theory Comput. **14**, 5834 (2018)

[2] G. Landrum, P. Tosco, B. Kelley, S. Riniker, Ric, gedeck, R. Vianello, N. Schneider, A. Dalke, D. N, B. Cole, M. Swain, S. Turk, D. Cosgrove, A. Savelyev, A. Vaucher, M. Wójcikowski, G. Jones, D. Probst, V.F. Scalfani, G. Godin, A. Pahl, F. Berenger, J.L. Varjo, strets123, JP, DoliathGavid, G. Sforna, J.H. Jensen, (2021)

[3] D.L. Mobley, J.P. Guthrie, J. Comput. Aided Mol. Des. **28**, 711 (2014)

[4] R. Wolfenden, Y.L. Liang, M. Matthews, R. Williams, J. Am. Chem. Soc. **109**, 463 (1987)

[5] J.P. Guthrie, J. Comput. Aided **28**, 151 (2014)

[6] R.C. Rizzo, T. Aynechi, D.A. Case, I.D. Kuntz, J. Chem. Theory Comput. **2**, 128 (2006)

[7] J.P. Guthrie, J. Phys. Chem. B **113**, 4501 (2009)

[8] A. Nicholls, D.L. Mobley, J.P. Guthrie, J.D. Chodera, C.I. Bayly, M.D. Cooper, V.S. Pande, J. Med. Chem. **51**, 769 (2008)
